# High-depth whole genome sequencing of premalignant breast lesions reveals rearrangement hotspots and personalized management opportunities

**DOI:** 10.1038/s41467-026-72952-1

**Published:** 2026-05-19

**Authors:** Lucia Chmelova, Helen R. Davies, Andrea Degasperi, Giuseppe Rinaldi, Leon Wils, Daniella Black, Yasin Memari, Gene C. C. Koh, Scott Shooter, Lennart Mulder, Petra Kristel, Karoly Szuhai, Gino Prasad, Ivy Tsz-Lo Wong, Jens Luebeck, Yogesh Kumar, Jan Czarnecki, Shadi Basyuni, Zoya Kingsbury, Mark T. Ross, Øystein Garred, Margit Riis, Karin T. Lande, Johan Vallon-Christersson, Anna Ehinger, Amit Agrawal, Stuart A. McIntosh, Vineet Bafna, Paul S. Mischel, Grand Challenges PRECISION Consortium, Fariba Behbod, Proteeti Bhattacharjee, Deborah Collyar, Helen Davies, Andrew Futreal, E. Shelley Hwang, Jos Jonkers, Esther H. Lips, Nicholas Navin, Serena Nik-Zainal, Donna Pinto, Daniel Rea, Elinor J. Sawyer, Marjanka Schmidt, Hilary Stobart, Alastair Thompson, Marja van Oirsouw, Jacco van Rheenen, Ellen Verschuur, Jelle Wesseling, Lodewyk F. A. Wessels, Jelle Wesseling, Johan Staaf, Therese Sørlie, Esther H. Lips, Serena Nik-Zainal

**Affiliations:** 1 Department of Genomic Medicine, University of Cambridge, Cambridge, UK; 2 Division of Molecular Pathology, The Netherlands Cancer Institute, Amsterdam, the Netherlands; 3 Sir Jeffrey Cheah Sunway Medical School, Faculty of Medical and Life Sciences, Sunway University, Sunway City, Malaysia; 4 Department of Cell and Chemical Biology, Leiden University Medical Center, Leiden, the Netherlands; 5 Department of Computer Science and Engineering, UC San Diego, La Jolla, CA US; 6 Department of Pathology, Stanford University School of Medicine, Stanford, CA US; 7 Sarafan ChEM-H, Stanford University, Stanford, CA US; 8 Illumina Cambridge Ltd., Granta Park, Great Abington, Cambridge, UK; 9 Department of Pathology, Division of Laboratory Medicine, Oslo University Hospital, Oslo, Norway; 10 Department of Cancer Genetics, Institute for Cancer Research, Oslo University Hospital, Oslo, Norway; 11 Division of Oncology, Department of Clinical Sciences Lund, Lund University, Medicon Village, Lund, Sweden; 12 Department of Clinical Genetics, Pathology and Molecular Diagnostics, Lund University Hospital, Lund, Sweden; 13 Department of Surgery, University of Cambridge, Cambridge, UK; 14 Department of Breast Surgery, Cambridge University Hospitals, Cambridge Biomedical Campus, Cambridge, UK; 15 Johnston Cancer Research Centre, Queen’s University Belfast Faculty of Medicine Health and Life Sciences, Belfast, UK; 16 Department of Pathology, Netherlands Cancer Institute, Amsterdam, the Netherlands; 17 Center of Early Cancer Detection, Netherlands Cancer Institute, Amsterdam, the Netherlands; 18 Department of Pathology, Leiden University Medical Center, Leiden, the Netherlands; 19 Division of Translational Cancer Research, Department of Laboratory Medicine, Lund University, Lund, Sweden; 20 Institute of Clinical Medicine, Faculty of Medicine, University of Oslo, Oslo, Norway; 21 University of Kansas Medical School, Kansas City, KS USA; 22 Patient Advocates in Research, Danville, CA USA; 23 The University of Texas MD Anderson Cancer Center, Houston, TX USA; 24 Department of Surgery, Duke University School of Medicine, Durham, NC USA; 25 Division of Molecular Pathology, Oncode Institute, The Netherlands Cancer Institute, Amsterdam, the Netherlands; 26 DCIS411, San Diego, CA USA; 27 University of Birmingham, Birmingham, UK; 28 School of Cancer and Pharmaceutical Sciences, Faculty of Life Sciences and Medicine, Guy’s Cancer Centre, King’s College London, London, UK; 29 Independent Cancer Patients’ Voice, London, UK; 30 Department of Surgery, Dan L Duncan Comprehensive Cancer Center, Baylor College of Medicine, Houston, TX USA; 31 Borstkanker Vereniging Nederland, Utrecht, The Netherlands; 32 Division of Molecular Carcinogenesis, Oncode Institute, Netherlands Cancer Institute, Amsterdam, The Netherlands, Faculty of EEMCS, Delft University of Technology, Delft, Netherlands

**Keywords:** Breast cancer, Cancer genomics, Genomic instability, DNA damage and repair

## Abstract

Ductal carcinoma in situ is a non-obligate precursor lesion of breast cancer. Often detected by mammography, most cases are managed through surgical and/or radiotherapy approaches. Today, it is not possible to predict which patients will progress to invasive disease. Here, we evaluate high-depth whole-genome sequenced ductal carcinoma in situ, enriched for high-grade clinical lesions, to understand whether deep WGS could reveal biological insights and/or personalized therapeutic vulnerabilities that may be targetable. We find genomic locations that are likely susceptible to producing the initiating lesion for structural variations prone to subsequent evolution, termed SHOREs. We additionally highlight individualized therapeutic potential that would otherwise not be appreciable without whole genome sequencing. We posit that holistic whole genome sequencing profiling could offer a more precise stratification approach, discerning higher-risk cases for prospective clinical studies on personalized therapies, from truly low-risk cases suitable for active monitoring.

## Abstract (web summary)

Predicting progression from ductal carcinoma in situ (DCIS) to invasive breast cancer remains challenging. Here, deep whole genome sequencing of DCIS samples highlights possibilities for personalized medicine and identifies genomic sites, termed SHOREs, that may initiate structural variations leading to subsequent evolution.

## Introduction

Ductal carcinoma in situ (DCIS), a non-obligate precursor lesion of breast cancer (BC), represents 20–25% of newly diagnosed breast lesions^1^. The increase in incidence from 10 to 79 cases per 100,000 women driven by the implementation of screening mammography^2^ represents a major clinical challenge, as it is currently not possible to distinguish cases that will remain indolent from those that will progress. Therefore, almost all DCIS are treated with surgery with or without radiotherapy. Yet, 2–15% of women will still develop invasive BC despite surgical management^3^. On the other hand, small series of untreated DCIS have reported ipsilateral invasive recurrence rates at 10 years of 18–37% in high-grade versus 9–12% in low-grade DCIS^4,5^. This suggests that a large proportion of women with DCIS may be overtreated. Active surveillance has been proposed as a treatment de-escalation option, with multiple ongoing clinical trials, for low and intermediate-grade DCIS^6–8^. Thus, there is a pressing clinical need to identify factors that may increase the risk of recurrence to enable improved patient stratification for either active surveillance or treatment^9^.

Cancer initiation is dependent on a complex interplay of intrinsic and extrinsic factors – endogenous cellular abnormalities, the local tissue microenvironment, immunological dynamics, and environmental factors are anticipated to play contributory roles in promoting BC. In primary invasive breast cancer (IBC)^10,11^ and metastatic breast cancer (MBC)^12,13^, whole genome sequencing (WGS) has expansively uncovered the landscape of intrinsic genomic factors – causally implicated driver mutations, mutational signatures, structural abnormalities, and germline variation – that underpin these fully malignant states. However, a comprehensive appreciation of the genomic landscape of DCIS has not yet been undertaken.

Here, we use high-depth WGS to explore DCIS and find surprisingly early emergence of high levels of mutagenesis, particularly extensive structural rearrangements, revealing biological insights into genomic instability in BC evolution. Furthermore, we identify a plethora of potentially targetable therapeutic vulnerabilities in nearly half of DCIS lesions, highlighting individualized information that could be used to inform future clinical studies.

## Results

### DCIS have similar drivers, copy number aberrations, and mutational signatures to IBC

**Fig. 1:**
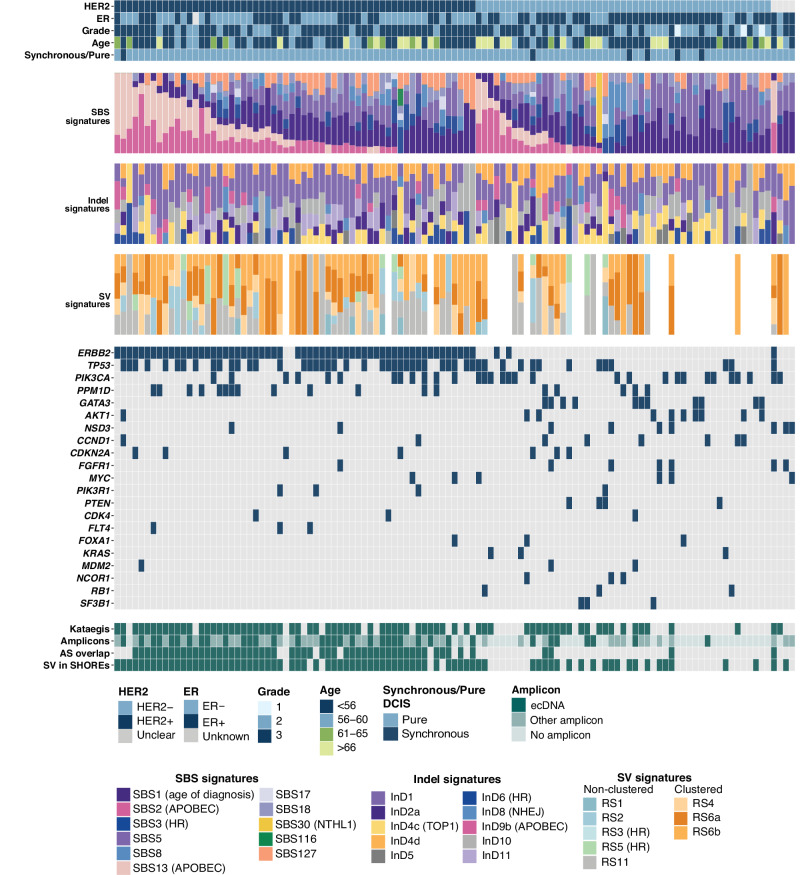
Genomic and clinical landscape of DCIS patients (*n* = 113). Each bar represents one of 113 DCIS lesions, ordered by HER2 status and decreasing proportional APOBEC mutational exposure (unassigned mutations not displayed). SBS, single base substitutions; SV, structural variation; SHOREs, Sites of Hotspot Rearrangements and Evolution; AS, Amplicon-SHORE. Genes were displayed if harboring ≥3 driver mutations in the cohort. Samples with SV count <25 did not have SV signatures assigned (white bar). Where not indicated by the legend, colored and gray bars indicate presence and absence, respectively. Source data are provided as a Source Data file.

A total of 113 paired DCIS and matched-normal samples were obtained from patients recruited at three European sites: the Netherlands, Norway, and Sweden (Supplementary Data 1). Compared to other reported DCIS populations^14–16^, this cohort (Fig. 1) was skewed towards high-grade lesions (63.7% versus 47.0–51.5%) due to limited available material for DNA extraction from low-grade lesions which tend to be smaller in size. This cohort also contains a higher proportion of younger patients ( < 56 years, 56.6% versus 31.8%) and HER2-positive lesions (53.1% versus 34.4%) (Fig. S1). Throughout the manuscript, we have contrasted the DCIS cohort to WGS data from 3186 IBCs^10,11^ and 661 MBCs^12,13^ when appropriate to the analysis involved.

Driver events in DCIS (Supplementary Data 2) were concordant with previous reports and similar to drivers identified in IBC^10,17^. The most frequent driver event was *ERBB2* (54.0%: 51.3% amplifications, 2.7% other) (Fig. 1, S2A). Other common drivers included *TP53* (43.3%), *PIK3CA* (28.3%), *GATA3* (11.5%), but also *AKT1*, *CCND1*, and *CDKN2A* (all <10%). Common copy number aberrations from GISTIC analysis included gains of 1q32.1, 8p11.23 (*NSD3, FGFR1*), 11q13.3 (*CCND1*), 17q12 (*ERBB2*), 17q23.2 (*PPM1D*), 20q13.2 (*ZNF217*) and the loss of 9p21.3 (*CDKN2A*), 17p12 (*MAP2K4*) (Fig. S2B, C) akin to observations in IBC and previous DCIS studies^10,17^.

Mutational signatures in DCIS showed similarity to IBC (Fig. S3A). Substitution (SBS) and indel signatures (InD) (Supplementary Data 3)^18,19^ associated with benign endogenous processes, including SBS1, SBS5 and InD1, were most prevalent (Fig. 1, S3B). These signatures tended to decrease in proportion as grade increased (Fig. S3C), consistent with the expectation that benign imprints would be dominant in earlier, pre-malignant lesions. Conversely, InD2a, associated with high proliferation markers in IBC^11^, became more prominent in high-grade samples (48%) (Fig. S3C), supporting previous reports of it being associated with a more malignant process. Other common mutational signatures in DCIS included APOBEC-related mutagenesis (SBS2, SBS13, or InD9). APOBEC mutagenesis represents a pathophysiological process involving substantial periods of transient single-strandedness of DNA (ssDNA), especially in hypermutated cases^20^. We detected APOBEC-associated signatures in 65% of DCIS samples, with 19.4% showing both SBS and InD APOBEC mutagenesis (Fig. S3D). The correlation between SBS and InD APOBEC signatures was weaker in DCIS than previously reported in IBC (Kendall correlation, τ = 0.32, *P* = 3.28×10⁻⁵)^11^. In terms of burden and prevalence, APOBEC SBS2 and SBS13 signatures increased across DCIS grades, and were generally comparable with IBC (Fig. S3E, F). By contrast, InD9 was much less prominent in DCIS (24% of samples) compared to IBC (49%) and MBC (42%), staying uniformly low across grades (Fig. 1, S3A, E). This suggests a later onset of APOBEC indel-mutagenesis in BC evolution. The disparity reflects mechanistic differences reported previously between SBS and indel APOBEC signatures, despite their occurrence at the identical sequence context^11^. Five DCIS lesions exhibited InD9 without SBS-APOBEC (Fig. S3D), including a sample with hypermutation of InD9 (LP2102411, 230 indels, 29%). Clusters of localized APOBEC substitution hypermutation termed kataegis were identified in 64.6% of DCIS samples (Fig. 1), comparable to 62.5% in high-coverage IBC (*n* = 2445)^11^. However, within samples, kataegis was more extensive in IBC where 13.9% tumors contained ≥10 kataegis loci, compared to DCIS, where only one sample ( < 1%) exhibited 10 kataegis loci.

Together, these findings demonstrate that the mutational and CNA landscape in DCIS strongly resembles that of IBC, with APOBEC-mediated processes and HER2-driven amplification already established in pre-invasive disease.

### Clustered structural variant signatures are strikingly enriched in DCIS and correlate with focal amplicons

**Fig. 2:**
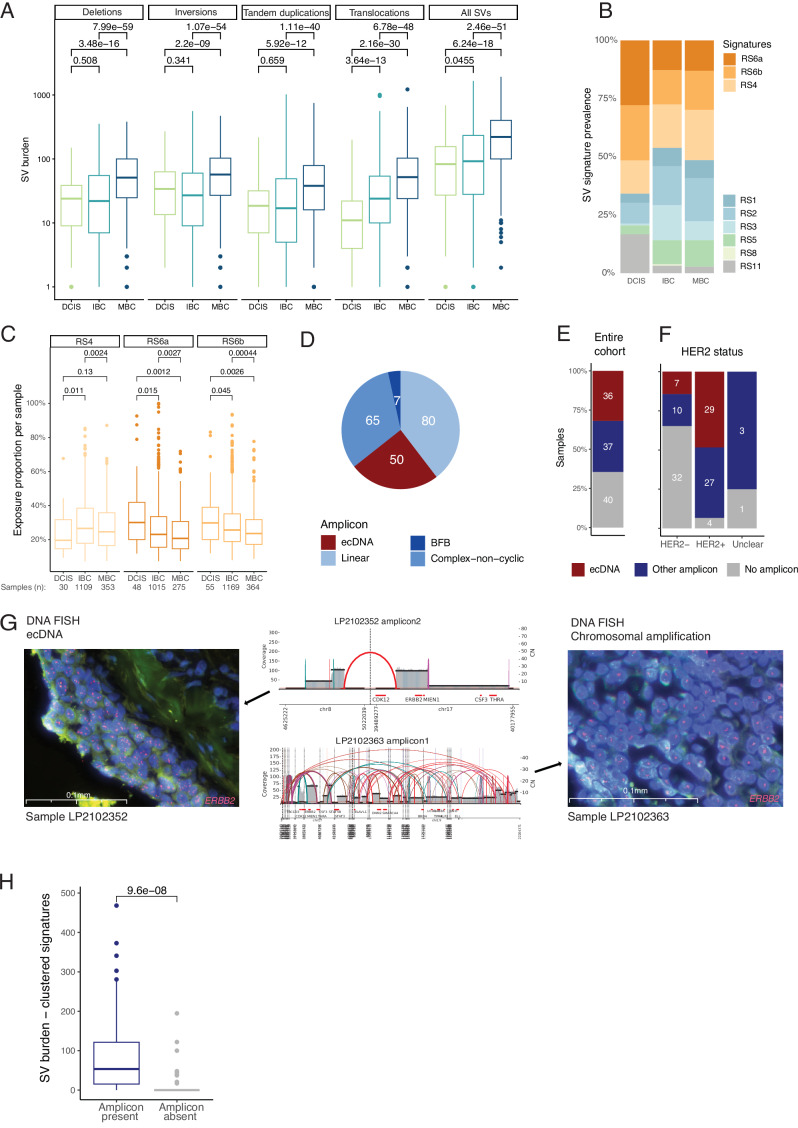
Clustered SVs and amplicons characterize DCIS. **A** SV burden by rearrangement type across DCIS (*n* = 113), IBC (*n* = 3186), and MBC (*n* = 661). *n* – number of samples. Wilcoxon rank-sum two-sided test. **B** Prevalence of aggregated RS exposure across BC stages. DCIS (*n* = 113), IBC (*n* = 3186) and MBC (*n* = 661). *n* – number of samples. **C** Comparison of exposure proportion of clustered RS per sample across BC stages, including only samples with exposure > 0. Two-sided *t*-test. **D** Distribution of amplicons (*n* = 202) in DCIS samples by type as predicted by AmpliconSuite^21^. **E**,**F** Distribution of samples (*n* = 113) based on the type of amplicon present, stratified by the entire cohort (**E**) and HER2 status (**F**). **G** Examples of *ERBB2* amplified via ecDNA (left) and complex-non-cyclic chromosomal amplification (right) confirmed by DNA FISH images (two representative samples from a total of 9 samples subject to FISH analysis). Red, *ERBB2* prob; green, chr17 centromere, 0.1 mm scale bar in the bottom left-hand corner of each image. **H** Clustered SV burden (RS4, RS6a, RS6b) in samples with amplicon (*n* = 76 samples) and without amplicon (*n* = 37 samples). Wilcoxon rank-sum two-sided test. Boxplots (A,C,H) show the median, 25–75th percentiles, with whiskers extending to ±1.5 × IQR (the interquartile range). Source data are provided as a Source Data file.

Structural variants (SVs) were surprisingly prevalent in DCIS with burdens rivaling IBC for all classes (*P* > 0.05, Wilcoxon signed-rank test) except translocations (Fig. 2A). Compared to IBC, DCIS showed significant enrichment for rearrangement signatures (RS) that are clustered, particularly RS6a (42.5% vs 31.8%, χ^2(1)^ = 5.1597, *P* = 0.02312) and RS6b (48.7% vs 36.7%, χ^2(1)^ = 6.2088, *P* = 0.01271), but not translocation-specific RS4 (26.5% vs 34.8%, χ^2(1)^ = 2.9384, *P* = 0.0865)(Fig. S4A, B, Supplementary Data 3). RS6a and RS6b formed a greater proportion of total rearrangements in DCIS than in IBC or MBC (Fig. 2B, C).

To explore further, we applied AmpliconSuite, an algorithm that reconstructs focally amplified regions to infer their structure^21^. 202 amplicons were identified across 76/113 (67.2%) samples, with 50 (24.8%) amplicons predicted to be extrachromosomal DNA (ecDNA) (Fig. 2D, Supplementary Data 4). Of note, 31.8% (36/113) of DCIS samples in our high-coverage WGS cohort skewed towards aggressive DCIS harbored at least one ecDNA-derived amplicons (Fig. 2E), substantially higher than reported previously ( < 10%) using shallow WGS^22^. ecDNAs were present in 48.3% (29/60) of HER2-positive cases (Fig. 2F), akin to HER2-positive IBC (46.4%)^23^. *ERBB2* was amplified via ecDNA in 20 (30%) HER2-positive samples and associated with significantly higher CN (Fig. S4C, Supplementary Data 5). *ERBB2* amplification status predicted by AmpliconSuite was confirmed in 8/9 samples by DNA FISH analysis (Fig. 2G). While focal amplicons strongly characterized HER2-positive DCIS present in 93.3% samples (Fig. S4D), 34.7% (17/49) HER2-negative DCIS samples also contained a cyclic or non-cyclic amplicon, highlighting that amplicon formation was not solely driven by *ERBB2* amplification.

The presence of amplicons is associated with clustered signatures (RS4, RS6a, and RS6b) (Fig. 2H). This high prevalence of amplicons in DCIS explains the enrichment of clustered signatures compared to IBC and MBC, which have also had a longer cellular time and greater opportunity for other non-clustered rearrangement processes to influence their genomic landscapes. These results demonstrate that focal amplifications and ecDNA formation are common early events in DCIS, tightly linked to clustered SVs.

### Focal amplicons are evolutionary accelerants and dependent on TP53 disruption

Amongst HER2-positive samples (*n* = 60), SV burden was comparable between intermediate and high grade, unlike in HER2-negative samples (Fig. S4E). Amplicon characteristics mirrored this pattern: in HER2-positive samples, amplicons were similarly present in intermediate- (100%) and high-grade (93%) cases, showing equivalent complexity score (Fig. S4D, F, Supplementary Data 4). Conversely, amongst HER2-negative samples, amplicons were less common but increased in prevalence and complexity (*P* = 0.0025, *t*-test) between intermediate and high grade (Fig. S4D, F), while carrying oncogenic drivers such as *CCND1*, commonly co-amplified*FGFR1* and *NSD3*, and *EGFR*. These data hint at genomic instability arising early in pre-malignant breast lesions, in the case of *ERBB2* accelerating very early, dominating the landscape of even intermediate-grade DCIS, while other driver amplicons likely evolve and drive progression later in DCIS development.

ecDNAs enable high levels of oncogene transcription and sometimes carry immunomodulatory genes that may contribute to immune cell infiltration and evasion of immunity^23–25^. However, in five ecDNAs (4/5 in ER-HER2+ samples) there was no evidence of oncogenes or immunomodulatory genes on ecDNAs, implying that formation and retention of ecDNA in pre-invasive lesions may not necessarily be oncogene-driven. Disruption of *TP53* pathway genes, including p53-repressors*PPM1D*, *MDM2*, and *MDM4*, was associated with amplicon presence (*P* < 0.0001, Fisher’s exact test) (Fig. S4G), broadly in agreement with previous observations^26^.

### Nexuses of susceptibility to breaks for all classes of SVs

**Fig. 3:**
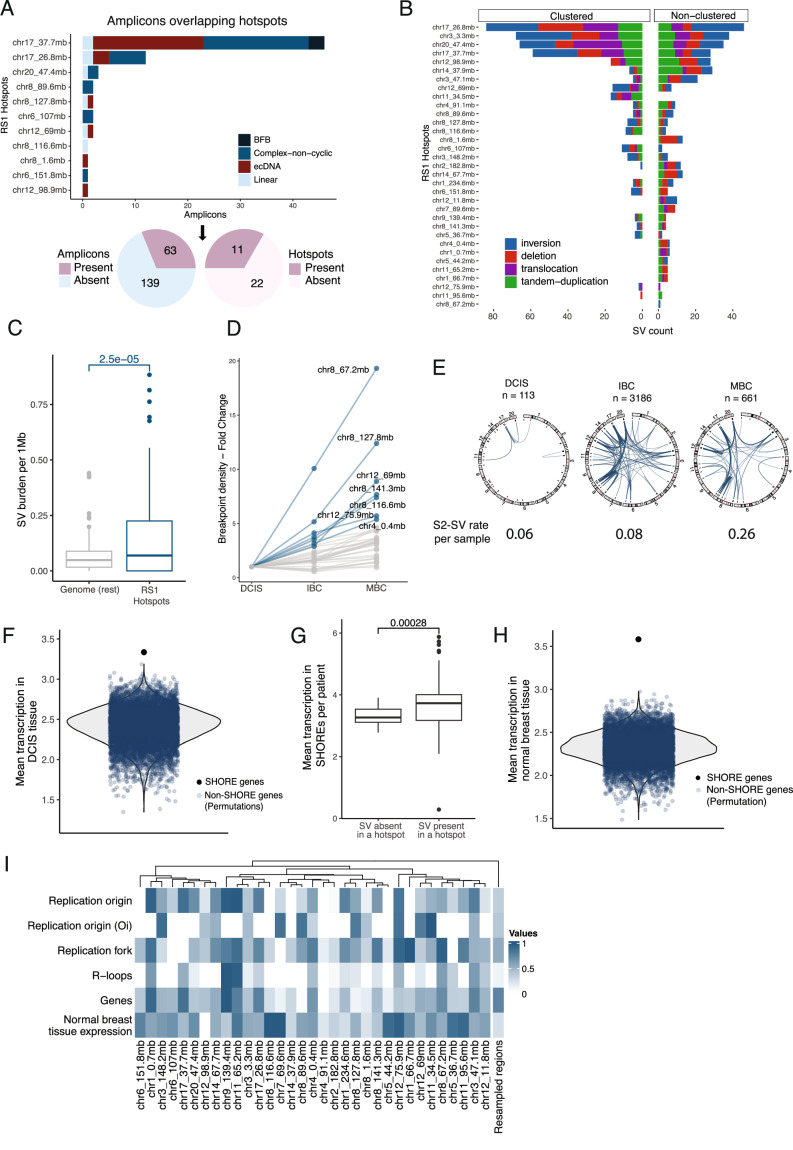
SV hotspots (SHOREs) are fragile loci driving ongoing DCIS genome instability. **A** Amplicons overlapping with RS1 hotspots in DCIS (63/202 amplicons; 11 hotspots). **B** Structural variants (SV) within RS1 hotspots in DCIS samples (*n* = 113) based on the SV clustering determined for each sample separately. **C** Density of SVs in DCIS (*n* = 113 samples) within RS1 hotspots compared to the rest of the genome. Wilcoxon paired signed-rank two-sided test. **D** Fold change in the breakpoint density by RS1 hotspots across DCIS (*n* = 113), IBC (*n* = 3186) and MBC (*n* = 661). *n* – number of samples. **E** SHORE-to-SHORE (S2) SVs with both breakpoints within RS1 hotspots across BC stages (DCIS: 7 S2-SVs, *n* = 113 samples; IBC: 254 S2-SVs, *n* = 3186 samples; MBC: 177 S2-SVs, *n* = 661 samples). Features depicted in the circos plots from outwards: karyotypic ideogram, SHOREs regions, and central lines represent S2-SVs with both breakpoints within SHOREs. S2-SV rate is an average number of S2-SVs per sample for each BC stage. **F** Mean transcription rate within SHORE genes compared to the mean transcription across 10,000 randomly selected sets of non-SHORE genes of the same size gene count (Permutation test, *n* = 10,000; for each sample) in DCIS (*n* = 97). **G** Paired comparison of mean transcription rate in SHOREs harboring SV versus in SHOREs without SV in the same sample (*n* = 97 samples). Wilcoxon paired signed-rank two-sided test. **H** Mean transcription rate within SHORE genes compared to the mean transcription across 10,000 randomly selected set of non-SHORE genes of the same size gene count (Permutation test, *n* = 10,000; for each sample) in normal breast tissue samples (*n* = 514). (**I**) Normalized density of genomic features within individual SHORE regions (*n* = 33) and randomly resampled SHORE-like regions (*n* = 33): replication origins, oncogene-induced (Oi) replication origins, replication forks, r-loops, protein-coding genes; and normal breast tissue expression (*n* = 514 samples). Boxplots (C,G) show the median, 25th–75th percentiles, with whiskers extending to ±1.5 × IQR (the interquartile range). Source data are provided as a Source Data file.

Given the very early onset of amplicons preceding carcinogenesis, we explored their possible origins. We utilized previously reported hotspots of rearrangements driven by a long ( > 100 kb) non-clustered tandem duplication (TD) signature, RS1^27^. RS1 TD-hotspots are genomic regions previously shown to be enriched for tissue-specific super-enhancers (SE) (OR = 3.54 for RS1), BC germline predisposition SNPs (OR = 4.28), and were suggested as sites of selective susceptibility prone to DNA damage leading to breaks^27^. Thus, we asked whether amplicons overlapped with RS1 hotspots. We found 31% (63/202) of amplicons overlapping with 33% (11/33) of RS1 hotspots (Fig. 3A, S5A, Supplementary Data 6). The amplicons overlapping with RS1 hotspots were not restricted to ecDNA – 60% included non-ecDNA amplicons (Fig. 3A). Indeed, two ecDNAs and four non-ecDNA amplicons without oncogenes overlapped with RS1 hotspots, raising the possibility that RS1 hotspots had a role in initiating amplicons irrespective of selection.

To investigate susceptibility to breaks further, we asked whether all RS1 hotspots (and not just the ones under selective pressure to evolve into amplicons) could be sites of an initiating lesion/break for SVs. Indeed, we found that every RS1 hotspot (100%) showed at least one SV in this DCIS cohort (Fig. 3B, S5A, Supplementary Data 7). RS1 hotspots had higher density of SVs than the rest of the genome (*P* = 2.5e-05, paired Wilcoxon signed-rank test, Fig. 3C), representing a 3.17-fold enrichment in breakpoint rate (95% CI: 1.55–5.80, bootstrap test, *n* = 1000) (Fig. S5B). More than 2-fold enrichment was observed in 13 regions, the highest 37.5-fold enrichment was observed for chr17_37.7 mb (driver: *ERBB2*) (Fig. S5C). Although hotspots were first identified as sites of recurrent long TDs, only a small proportion (6.2%, 7/113) of DCIS samples harbored a TD-driven RS1 signature, suggesting that the TD-based mutational process was not a prerequisite for the initiation of DSBs (Fig. 3B, S5D). Indeed, a wide range of SV-types were present across hotspots and samples.

### Hotspots of SVs undergo on-going evolution

Ten amplicons (6 complex-non-cyclic, 3 ecDNAs, 1 linear) included multiple RS1 hotspots (Fig. S5A, Supplementary Data 6), fueling the notion that these are sites that not only break early in tumor development, but they also fuse, and subsequently co-amplify. To seek further evidence of ongoing evolution, we assessed the prevalence and density of breakpoints in RS1 hotspots across the spectrum of breast evolution. Seven hotspots showed increasing breakability from DCIS to IBC and MBC (fold change > 5) (Fig. 3D). Intriguingly, while the well-known chr8_127.8 mb (driver: *MYC*) hotspot showed increasing breakability from DCIS through to MBC, another hotspot chr8_67.2 mb showed the biggest fold change, having only single breakpoints in DCIS lesions, undergoing very marked evolution as breast lesions become more aggressive.

These data imply that not only are these loci susceptible to breaks with ensuing SV formation, but they can also subsequently come under strong selection to form complex focal amplicons, ecDNA or otherwise, and can variably evolve further through IBC and MBC. Given these observations, we have renamed the RS1 TD-hotspots as Sites of Hotspot Rearrangements and Evolution (SHOREs).

We further observed simultaneous breakability of seven SHOREs in six samples, where SV breakpoints joined two different SHOREs. We termed them S2-SVs (SHORE-to-SHORE SVs) (Fig. 3E, Supplementary Data 7). This phenomenon can only manifest if both sites had a break at the same timepoint in the same cell cycle. The occurrence of S2-SVs represents a significant enrichment compared to random resampling of SVs (*n* = 101, *p* = 0.0098). We next assessed the presence of S2-SVs in IBCs and MBCs. We identified 254 S2-SVs in IBC, impacting all 33 SHOREs (Fig. 3E). Interestingly, 177 S2-SVs detected in the MBC correspond to >4-fold higher rate of S2-SVs formation compared to IBC (Fig. 3E), reinforcing the notion of general breakability of SHOREs.

The most common intrachromosomal S2-SV pairs were chr17_26.8mb–chr17_37.7 mb (DCIS: *n* = 2, IBC: *n* = 26; MBC: *n* = 7) and chr8_116.6mb–chr8_127.8 mb (DCIS: *n* = 0; IBC: *n* = 23; MBC: *n* = 10), respectively. The most common inter-chromosomal S2-SV pair was chr12_69mb - chr20_47.4 mb found in 9 samples overall. In total, there were 4 (57%), 140 (55%) and 119 (67.2%) inter-chromosomal S2-SVs (translocations) in DCIS, IBC, and MBC, respectively.

SHOREs with the most S2-SVs are chr17_37.7 mb in IBC (1.3% samples) and chr8_116.6 mb in MBC (2.5% samples). While chr17_37.7 mb also exhibited concurrent fragility in DCIS, chr8_116.6_mb was not affected by any S2-SVs and showed only a limited number of SVs overall in DCIS. This suggests that the chr8_116.6 mb region, encompassing *TRPS1*, a gene previously implicated in breast cancer^28^, may be a site of structural vulnerability only during invasive progression rather than in DCIS.

Finally, we examined the clonal characteristics of the SVs in SHOREs across the patient cohort to find that ten SHOREs including chr17_37.7 mb (*ERBB2*) harbored only clonal characteristics (Fig. S6A) and no SHOREs were found to be exclusively subclonal. These results reinforce how SVs at SHOREs are early contributors to tumor evolution.

### Transcription increases the likelihood of breaks in SHOREs

To investigate why SHOREs are susceptible to breaks in general, we explored several potential mechanisms. A known source of physiological breaks is high transcriptional activity^29^. First, to assess transcription levels in SHOREs, RNA-seq data available for 97 DCIS samples were utilized. We found that genes within SHOREs had significantly higher expression than a randomly selected set of non-SHORE genes with a similarly sized genomic footprint (10,000 permutations, *P* = 0.0001; Fig. 3F). Second, we contrasted SHOREs that harbored at least one SV to those in the same sample that had no SVs and found that the former had significantly greater mean transcription compared to SHOREs without SVs, regardless of SV type (*P* = 0.0027, pairwise Wilcoxon signed-rank test, Fig. 3G, S6B). This supports the notion that high hotspot transcription contributes to break formation. Third, to confirm that transcription contributes to genome instability in premalignant tissues, we sought transcription rates in normal non-cancerous breast mammary tissue samples (*n* = 514) obtained from adult Genotype Tissue Expression (GTEx) Project^30^. Indeed, SHORE genes showed a higher mean transcription rate than randomly selected non-SHORE genes (permutation test, *P* = 0.0001) (Fig. 3H, I). Additionally, some of these genes are likely breast-specific effects, as seven hotspot genes (*CIDEC*, *ELF5*, *OXTR*, *SEMA3G*, *SPINK8*, *TRPS* and *TTC6*) were previously identified as having elevated expression in breast only compared to other tissues based on the Human Protein Atlas results^31^. This emphasizes the role played by tissue-specific transcription in shaping the SV landscape in breast tissues from a pre-malignant state.

### Replication and transcription conflicts may be the determinant of breaks

To understand why some SHOREs were more susceptible to rearrangements than others, we considered the potential role of transcription-replication conflicts (TRCs). Regions with both high transcriptional activity and a dense presence of origins of replication (OR) are more likely to experience collisions between transcription and replication machinery that can lead to replication fork stalling and, if unresolved, the generation of breaks. Using OR maps^32^, SHOREs were found to be significantly enriched in replication origins compared to randomly resampled regions (27/33, *P* = 0.009, Fig. 3I, S6C, Supplementary Data 8).

Co-localization of ORs with sites of higher transcription could increase the likelihood of TRCs and consequentially result in stalled forks and the formation of R-loops (RNA-DNA hybrids)^33^. In support of this postulate, we found that using BrdU peak maps as a proxy for fork stalling^34^, significant enrichment of replication fork stalling was noted within SHOREs (29/33; *P* = 0.031; Fig. 3I, S6C). SHOREs were also found to be significantly enriched in R-loops (*P* < 0.001; Fig. 3I, S6C), where variable densities were observed across SHOREs, with some regions e.g., chr9_139.4 Mb and chr11_65.2 Mb showing especially high R-loop densities.

Together, these findings suggest that SHOREs are likely to be sites of elevated TRCs, creating a high-risk locale for breaks. These can manifest as isolated or clustered rearrangements and may initiate amplicons, including ecDNAs, even at pre-cancerous (in situ) stages of breast cancer. The RS1 signature central to the SHOREs original identification was also prevalent in the MBC (20.5%) (Fig. S4B), confirming the continued vulnerability of these regions, where early intervention may prevent acceleration of regional breakability.

### Individualized features in DCIS: value of WGS

**Fig. 4:**
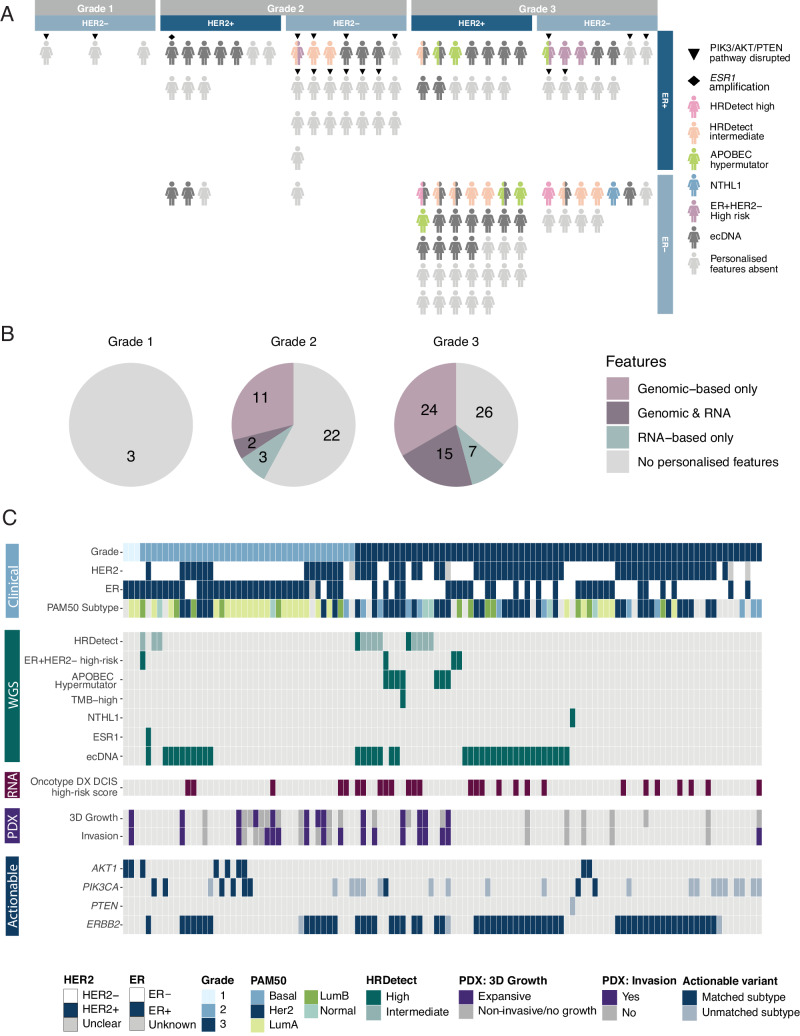
WGS reveals individualized and clinically actionable features in DCIS. **A** Schematic representation of DCIS patients with a known ER and HER2 status (*n* = 108) colored by the WGS-derived composite personalized features. Notable genomic mutations (disruption of PIK3/AKT/PTEN pathway and *ESR1* amplifications) are indicated. **B** Proportion of patients (*n* = 113) with individualized features, stratified by Grade and NGS feature type. Genomic-based features include the WGS-derived features from (**A**), RNA-based features are based on OncotypeDx classification (available for 97 patients). **C** Overview of clinically relevant features present in DCIS patients (*n* = 113). APOBEC hypermutator was defined as having a combined exposure of SBS2 and SBS13 > 80% of total SBS exposure. Patients are ordered by grade and cumulative number of WGS-based features. TMB, tumor mutational burden; PDX, patient-derived xenograft. Where not indicated by the legend, colored and gray bars indicate presence and absence, respectively. Source data are provided as a Source Data file.

Finally, we asked whether WGS could uncover clinically relevant features in DCIS with personalized medicine potential. We integrated information from drivers, mutational signatures, compound algorithmic scores (HRDetect, TMB) and other omic modality risk predictions, including transcriptomic assays where available (e.g., PAM50, Oncotype Dx^35^) (Fig. 4). We included information on 3D growth of patient-derived xenograft (PDX) models^36^ for a subset of samples (*n* = 32). Below we highlight several instances where there is currently no targeted management for DCIS but where a genomic feature could influence clinical decisions or have immediate clinical trial opportunities.

Independent of HER2 status, we identified 52/113 cases with WGS features that could potentially influence clinical outcome in the future: HRDetect high/intermediate scores, WGS-based prognostic factors in ER-positive HER2-negative (ER + HER2-) IBC^11^, APOBEC hypermutator status, biallelic *NTHL1* mutations, *ESR1* amplifications, and ecDNA. 35/52 (67%) samples were not detected by transcriptomic-based predictors (Fig. 4A, B). Conversely, RNA-based classification identified ten cases as high-risk, but no genomic features of concern were apparent (Fig. 4B).

One high grade ER-HER2- DCIS patient (LP2101807) carried a constitutional pathogenic *BRCA1* mutation with LOH of the wildtype allele (Fig. 4, S7), and had the associated mutational signatures, a clear HRDetect score of 0.99 indicating HR-deficiency^37^. One ER-HER2+ high-grade case (LP2102366) with high HRDetect score (0.94) (Fig. S7) exhibited InD6, RS5, SBS3, SBS8 signatures analogous to HR-deficient samples. However, it also showed an InD4c hypermutator phenotype (InD4c exposure: 994, 46%, attributed to transcription-associated mutagenesis)^11,19,38^, and lacked a *BRCA1/BRCA2* mutation. Another InD4c hypermutator ER-HER2- case (LP2101811, InD4c exposure: 1827) had an intermediate HRDetect score (0.72) and showed expansive PDX 3D-growth and invasion. We have previously suggested that this InD4c-driven phenotype might have alternative etiologies and therapeutic vulnerabilities, and warrants further pre-clinical and clinical investigation^19^.

Seven other high-grade samples (9.7%) also had intermediate HRDetect scores (0.1 − 0.9) (Fig. 4). Four of these had a PDX model available – all showed PDX invasion, and three also showed expansive 3D growth. Three were ER-HER2- akin to the poorest surviving TNBC HRDetect-intermediate group reported previously^39^. Another seven high-grade samples displayed APOBEC hypermutator phenotypes ( > 80% exposure, four samples also harbored InD9) (Fig. 4), highlighting early emergence of a phenotype that is otherwise enriched in MBCs (16.6%) and constitutes a high-risk group in IBC^11^. One patient with an ER-HER2- lesion was a constitutional heterozygous carrier of *NTHL1* pathogenic mutation with somatic loss of the second allele. The deficiency of the *NTHL1* DNA glycosylase involved in base excision repair manifests as high SBS30 exposure. While heterozygous *NTHL1* variants have not been associated with increased breast cancer risk, the complete loss of *NTHL1* function may confer a distinct biological vulnerability and potential risk of recurrence as IBC^40^. These are lesions for which precision medicine clinical trials would potentially be a suitable strategy.

Amongst intermediate-grade cases, three were HRDetect intermediate and ten harbored ecDNA associated with poor prognosis in IBC^24^. Five of these were high-risk based on DCIS-specific RNA-based predictors. Overall, relevant genomic and/or high-risk transcriptomic features were found in 63.9% of high-grade and 44.7% intermediate-grade samples.

Finally, we highlighted variants reported as clinically actionable in IBC (Fig. 4C). 17 ER+HER2- cases, including low and intermediate-grade and HRDetect-intermediate cases, had variants in the PIK3CA/AKT/PTEN pathway (*AKT* p.E17K, *PIK3CA* primarily p.H1047R, *PTEN* p.G127E), which are associated with response to Capivasertib and Fulvestrant in locally advanced ER+HER2- IBCs^41^. Five had relevant genomic and/or high-risk transcriptomic features (Fig. 4C). Additionally, *ERBB2* amplifications and point mutations targetable by Neratinib, Trastuzumab and Lapatinib in HER2-positive IBC were found in 58 HER2-positive DCIS patients – 40/58 had other individualized WGS/RNA features mentioned above. These included a sample with high TMB (14.3 mut/Mb), which could be eligible for immunotherapy in IBC, and an ER + HER2+ case (LP2102331) that harbored an *ESR1* amplification, a harbinger of endocrine therapy resistance^42^. However, ER and HER2 testing are currently not routinely performed in the diagnosis of DCIS^43^, restricting the clinicians’ ability to stratify patients with more aggressive lesions.

## Discussion

In this large high-depth WGS DCIS dataset, we find that DCIS are highly genomically altered lesions, harboring similar molecular makeup to IBC. There are, however, notable differences that we comprehensively dissect to understand breast tumor biology and clinical utility.

First, pre-malignant lesions provide a unique lens by which to view early genomic instability, revealing the initiating regions that are susceptible to rearrangements, or SHOREs. This is afforded by a clearer genomic canvas than IBC or MBC, unsullied by the complicating mutational processes that arise later in tumorigenesis. We find that SHOREs are susceptible sites that can produce any type of rearrangement, likely induced by transcription-replication conflicts, evidenced by high transcription rate in replication origin-enriched regions of normal and pre-cancerous breast tissue. Moreover, some SHOREs break simultaneously during a cell cycle, resulting in directly joining breakpoints or SHORE-to-SHORE SVs, underscoring the high level of breakability of these sites in normal breast cells. Importantly, SHORE fragility is not static. While many SHOREs exhibit early instability, others display marked increases in breakage across cancer evolution, consistent with progression-driven positive selection. In some lesions this leads to focal amplicon formation, including ecDNAs, which act as early evolutionary accelerants in a subset of HER2-positive DCIS. However, longitudinal studies are required to understand whether structural variants in SHORES could serve as a biomarker and harbinger of recurrence as IBC.

Second, we show DCIS is molecularly diverse. WGS revealed that 46% of lesions harbored targetable abnormalities and/or carried negative prognostication indices in IBC, including *ERBB2* amplified via ecDNA, HRDetect-high lesions, APOBEC hypermutation, *PIK3CA*/*AKT*/*PTEN* alterations, and NTHL1-driven base excision repair deficiency. This raises the question of whether a personalized, holistic approach may provide interventions that benefit specific patients more effectively. Conversely, we also identified cases that could be considered truly low risk for treatment de-escalation rather than surgery. This highlights the clinical potential of WGS to accurately discern cases for both categories.

Limitations of this study include the lack of systematic long-term follow-up restricting our ability to use these genomic features for prognostication of recurrence as IBC. The skew towards more aggressive lesions is not representative of the general DCIS landscape. However, these are the lesions that represent the clinical conundrum, where surgical intervention based on size, grade or multifocality occurs, but for which subsequent clinical management remains unclear. Furthermore, 44.7% of intermediate-grade samples (14 ER-positive, 3 ER-negative) harbored relevant genomic and/or high-risk transcriptomic features potentially increasing the risk of progression. Thus, the data provide insights that underscore the limitations of the histological grading in capturing the biological complexity of DCIS, echoing prior concerns about the reproducibility and prognostic utility of grading, especially for intermediate lesions, where disconcordance was highest^44^. Intriguingly, the extent of genomic instability observed even in pre-malignant lesions does raise a pertinent question – what is the tipping point at which a genomically-abnormal lesion truly becomes malignant? To address this question, we will indeed require a longitudinal view reaching beyond genomics into multimodal integration with (at least) the epigenetic and immunological spheres.

There is increasing interest in non-surgical treatment approaches for DCIS. The LORD-trial investigates whether it is safe to omit treatment altogether in women with low-risk DCIS^6^. In contrast, the CALGB40903 study looks at letrozole pre-operatively^45^, the COMET study aims to investigate tamoxifen in a subgroup analysis^46^, and the DCIS: RECAST study intends to look at various agents including a selective estrogen receptor degrader (SERD) and an androgen receptor agonist in DCIS, regardless of grade^47^. What our work provides is the genomic path to select patients for cost-efficient stratification into clinical trials. While we may swap the morbidity of surgery with the side effects of chemotherapy, some patients may consider this an acceptable trade-off given that what patients want from their treatment and what they want to de-escalate varies hugely^48^. We acknowledge that further work is required to validate these clinical propositions, and to develop the evidence base for these genomic markers in DCIS. Hearteningly, as DCIS is increasingly diagnosed using large-volume needle core biopsies, the possibility of integrating genomic assessment into clinical workflows is becoming increasingly likely.

## Methods

### Participants and samples

The DCIS cohort compromised 113 DCIS lesions recruited via three collection studies: Oslo University Hospital, Norway; Netherland Cancer Institute (NKI), the Netherlands; Lund University, Sweden. The use of the samples was subject to written patient consent and ethical approval of the respective collection center and this research complies with the relevant ethical regulations. Gender was not a consideration in the study design.

Patients in the Lund cohort were enrolled in the SCAN-B study (NCT02306096) during 2010–2014^49,50^. Ethical approval was given for the SCAN-B study (Registration numbers 2009/658, 2010/383, 2012/58, 2013/459, 2014/521, 2015/277, 2016/541, 2016/742, 2016/944, 2018/267, 2019/01252, and 2024-02040-02) by the Regional Ethical Review Board in Lund, Sweden, governed by the Swedish Ethical Review Authority, Box 2110, 750 02 Uppsala, Swede (registration numbers 2019-01252, 2024-02040-02). All patients provided written informed consent prior to enrollment, including to publish information about sex and age. All analyses were performed in accordance with patient consent and ethical regulations and decisions. Inclusion and exclusion criteria for SCAN-B patients are outlined in NCT02306096^49,50^.

The DCIS samples from Oslo were collected as part of the Oslo2 breast cancer observational study following written informed consent. The Regional Committee for Medical and Health Research Ethics for southeast Norway has approved the study (approval numbers 2016/433 and 2019/657).

The NKI sample cohort was collected from DCIS patients treated at the Netherlands Cancer Institute between 2017 and 2022. Fresh frozen DCIS tissue pieces were collected at the pathology gross room. The study was approved by the Institutional Review Board NKI-AVL at the NKI under approval number CFMPB658.

Fresh frozen (FF) lesions were obtained from patients that underwent either a breast conserving surgery (26.5%) or mastectomy (73.5%) and the collected tissues were subsequently micro-dissected (68.1%) or macro-dissected (31.9%) (Supplementary Data 1).

DCIS was synchronous to adjacent IBC in 6 patients (HER2 + 1/6). PDX data^36^ (*n* = 32) included information on presence of growth, 3D growth and invasion.

Where relevant, DCIS genomic features were compared to the invasive (*n* = 3186) and metastatic (*n* = 661) breast cancer cohorts described previously^10–13^:

- International Cancer Genome Consortium (ICGC, genomic data, *n* = 741)^10^. The average sequence coverage for tumors and matched normals was 43× and 30×, respectively.
- Genomics England (100kGP, genomic data, *n* = 2445)^11^. The average sequence coverage for tumors and matched normals was 96× and 36×, respectively.
- Hartwig Medical Foundation BCs (genomic data, *n* = 661)^13^. The sequence coverage for tumors ranged from 90–100× for tumor samples and 30–35× for matched normal samples.

### Whole genome sequencing

WGS was carried out by Illumina Cambridge Ltd, UK. Short insert 450–500 bp libraries were prepared in accordance with Illumina protocols using either Illumina Truseq PCR free protocol or Truseq Nano kit with 5 PCR cycles, depending on the quantity of starting DNA available. 150 bp paired-end sequencing was performed using Hiseq X or Novaseq 6000 to achieve an average sequence coverage of 104.3× in tumors and 39× in matched normal controls from the same individual. The resultant reads were aligned to the reference human genome (GRCh38) using a Burrows–Wheeler Aligner, bwa mem (version 0.7.17-r1188 within dockstore-cgpmap v3.2.0). Paired tumor-normal bam files were interrogated for somatic mutations using the following algorithms, CaVEMan (Cancer Variants through Expectation Maximization) (1.13.15)^51^ for single-nucleotide substitutions, Pindel version 3.2.0^52^ for small insertions and deletions, BRASS 6.2.1^53^ for structural rearrangements, and ASCAT 4.2.1^54^ for copy number changes.

Additional post-processing filters were applied prior to downstream analysis, for CaVEMan ASMD > = 140, CLPM = 0, for BRASS variants a successful local realignment step indicated by assembly score <0 was required. Pindel filters include QUAL ≥ 250, REP < 10, NP > 0, PP > 0; the calls were further filtered against SNPs with frequency > 0.01 to reduce the false positive rate. SNP databases used for filtering were gnomAD (version 4.0 whole genome data, build: hg38), and 1000 Genome Project (version 1000g2015aug, build: hg38)^55^. ASCAT was used to call copy number variants and to provide estimates of average tumor ploidy and aberrant cell fraction. The algorithms used are contained in the following dockstore (dockstore-cgpwgs v2.1.1 available at https://quay.io/repository/wtsicgp/dockstore-cgpwgs).

### Driver mutation assessment

Somatic mutations present in known cancer genes^10,56,57^ were reviewed and mutations were deemed to be potential driver mutations if they were consistent with the type of mutations found in a particular cancer gene. These consisted of inactivating mutations in tumor suppressor genes (TSG) (including nonsense, frameshift, essential splice site mutations, recurrent missense and disruption of the gene by structural rearrangement) and recurrent mutations in oncogenes. Recurrent mutations were determined by reference to reported mutation frequency in the COSMIC database^56^. Homozygous deletions affecting TSGs and focal amplifications of oncogenes were detected using ASCAT copy number data.

Amplifications were considered if the copy number was ≥ 5 for diploid tumors (with ploidy <2.7n) or ≥ 9, for tumors with evidence of whole-genome duplication with ASCAT ploidy > 2.7n.

### GISTIC

GISTIC ver 2 (v2.0.23) analysis was performed using the ShixiangWang/install_GISTIC^58^. The default parameters were changed as described in Nik-Zainal et al. (2016)^10^.

### Mutational signature fitting

#### DCIS cohort

Mutational signature fitting for substitutions, indels, and SV was performed as described previously^18,19,59^. Briefly, 96-channel mutational catalog of substitutions with the trinucleotide context for each sample was constructed. Indels were categorized into 89-channel catalog considering type, length and context. SV were classified into 32-channel catalog based on their type, length and presence within a cluster.

SBS signatures were assigned using FitMS function (r-package signature.tools.lib v2.4.4^60^), with 200 bootstraps and error reduction of 20%, using reference signatures described previously^18^. The rare signature assignment was manually curated. Indel signatures were assigned using FitMS and breast-specific indel signatures extracted as described by Koh et al. (2024)^19^ and published in Black et al. (2025)^11^. Only indels <100 bp were used to construct catalogs and assign signatures to increase their assignment accuracy. SV signatures were assigned for samples with a minimum of 25 SVs, using a function Fit (signature.tools.lib v2.4.4).

#### Other BC cohort

The signature fitting for other BC cohorts (ICGC, 100kGP, and HMF) was performed as described for DCIS, unless stated otherwise. SBS signature fitting for 100kGP were performed with 25% error reduction. Prior to the catalog construction, indels in HMF were additionally filtered against SNPs with frequency > 0.01 located ± 5 bp away from the call position to reduce the false positive rate, using the same SNP databases as for DCIS indel filtering.

#### HRDetect

HRDetect algorithm (signature.tools.lib v2.4.4)^37^ was applied to predict HR deficiency based on the following parameters: the proportion of deletions overlapping microhomology, exposures of substitution signatures SBS3 and SBS8, rearrangement signature RS3 and RS5, and HRD-loh index. The algorithm was executed using default parameters, HRDetect-high and HRDetect-intermediate were defined as scores > 0.9 and 0.1–0.9, respectively.

#### APOBEC hypermutators

Samples with the combined exposure of SBS2 and SBS3 ≥ 80% of the total SBS exposure were classified as APOBEC hypermutators.

#### InD4c hypermutators

Samples with InD4c exposure ≥ 900 were classified as InD4c hypermutators.

#### Tumor mutational burden

TMB for WGS was calculated as a total number of somatic substitutions and indels identified per Mb of sequenced region, where the length of the sequenced region in hg38 was 3088 Mb^61^. Samples with TMB > = 10 Mut/Mb were classified as TMB-high.

#### Kataegis

Regions of kataegis were identified using findKataegis function (signature.tools.lib v2.4.4).

#### ER and HER2 status assignment

ER/HER2 status was inferred from immunohistochemistry (IHC) and WGS-derived evidence. IHC for ER and HER2 status was performed by the diagnostic pathology laboratories of the relevant collecting center using the standard routine practice of that center. Ready to use antibodies CONFIRM anti-Estrogen Receptor (ER) (SP1) Rabbit Monoclonal Primary Antibody (Roche) and VENTANA® anti-HER2/neu (4B5) Rabbit Monoclonal Primary Antibody (Roche) were used by all centers. IHC was available in 74.3% and 67.3% of the cohort, respectively. Where IHC was not available (*n* = 26), an RNA-based single-sample predictor (SSP) was used^62^. ER and HER2 status could not be determined in 1 and 4 patients, respectively, where data was either not available or for HER2 the two lines of evidence were not in agreement.

#### ecDNA and amplicon predictions

Amplicon Architect v1.3.r5^63^ was used to determine cyclic paths from focal amplifications predicted using CNVKit v0.9.10. AmpliconClassifier v1.2.1 was applied to classify the amplicons, including ecDNAs; using the following filters to prevent false positive findings: similarity score filtering, decomposition strictness = 0.25 and minimum cyclic copy count = 3. Where samples were classified according to AmpliconClassifier results; if a sample contained both ecDNAs and other non-ecDNA amplicons, it was labeled as an ecDNA sample.

Complexity score was calculated by AmpliconClassifier, as previously described^23^, capturing the diversity of the ecDNAs and other non-cyclic amplicons present. For nine samples, FISH images from FFPE tissue were successfully analyzed, using *ERBB2* red probe and green chr17 centromere probe (Agilent HER2 IQFISH kit).

#### Hotspots analysis

RS1 hotspots, further described as SHOREs, were obtained from Glodzik et al. (2017)^27^. The genomic coordinates were converted from hg19 to hg38 using Bioconductor package liftOver (v3.19)^64^.

#### Breakpoint density and risk quantification

SV density was compared inside and outside of the SHOREs, excluding the flanking region on each side equal to half of the SHORE length (the total excluded length = SHORE length).

To compare the rate of breakpoints in the SHOREs to the rest of the genome (background), the genome (hg38) was separated into 500 kb windows and windows that overlapped with SHOREs were omitted from the further analysis.

Adjusted breakpoint rate for each window/SHORE was calculated as

1 $${{{\rm{rate}}}}=\frac{b}{n\times s}$$

where *b* is a total number of breakpoints in each region, *n* is total number of samples in the cohort and *s* is the size of the region. The enrichment was calculated as the ratio of average SHORE rate to the average background rate. The variability was assessed through bootstrap resampling (*n* = 1000), where the enrichment for each iteration was recalculated and 95% confidence interval was obtained.

#### Hotspot breakability across BC stages

For each BC stage, the total number of SVs overlapping each SHORE was calculated and then normalized to the respective cohort size. Fold-change values for IBC and MBC were obtained by comparing their normalized rates to those of DCIS.

#### Resampling SVs

To estimate the likelihood of a random SV overlapping 2 SHOREs (S2-SV), SVs from DCIS were resampled 101 times using the resampleSV function from r-package topography.tools.lib (v1.0.0). For each resampled set, the number of S2-SVs overlapping SHOREs was calculated. Enrichment was defined as the proportion of resampled sets with an S2-SV count greater than the observed count in the real data.

#### Resampling regions

To obtain a set of random regions that have the similar characteristics and genomic footprint (size, chromosome) as the SHORE regions, we used a function resampleBedRegions from r-package topography.tools.lib (v1.0.0). The regions were not allowed to overlap.

#### Clonality

The clonality of SHOREs was estimated for individual SHORE regions that overlapping SV (250 regions across 74 samples). Clonality was inferred based on variant allele fractions of SNVs within each region, analyzed with the MutationTimeR algorithm (v1.00.2)^65^. Genome-wide clonality was estimated based as a proportion of subclonal SNVs relative to the total number of SNVs across the genome for each sample.

#### Genomic features enrichment

The enrichment of the genomic features was calculated using multipleCorrelations function from topography.tools.lib (v1.0.0), where reference entity was SHORE regions, and the query entity included replication origins and oncogene-induced replication origins maps obtained from Macheret & Halazonetis^32^, BrdU peak regions that estimate the replication fork stalling^34^ and r-loop zones (level 8) available at R-loopBase^66^.

### Gene expression analysis

#### DCIS Tissue processing and library preparation

The RNA expression data were available for 97 DCIS samples. The NKI and Oslo samples (*n* = 65) were processed and sequenced at the NKI, while the Lund samples (*n* = 32) were processed at Lund University. 10 µm sections were cut from FFPE blocks and were either manually microdissected (NKI and Oslo) or macrodissected (Lund) using a light microscope. For NKI and Oslo samples, prior to dissection, the slides were stained using toluidine blue. RNA extraction was performed using the AllPrep DNA/RNA FFPE Kit (Qiagen). Total RNA samples were converted to strand-specific libraries using TruSeq RNA Access Library Prep kit (Illumina, RS-301-2001/2), according to manufacturer’s instructions, protocol part #15049525 Rev. B. Lund samples were prepared as previously described^62^.

#### RNA sequencing

For NKI and Oslo samples, the libraries were sequenced with 54 bp paired end reads using a Novaseq instrument (Illumina). Low-quality bases and adapters were removed using cutadapt (version 4.9, RRID:SCR_011841). Reads were aligned to the human genome (Gencode release 47^67^) with STAR (version 2.7.11 d, RRID:SCR_004463), in two steps to share novel splice site detection between samples in the same batch. Quality control was performed with FastQC (version 0.12.1, RRID:SCR_014583), Picard (CollectRnaSeqMetrics, version 3.2.0, RRID:SCR_006525), Samtools (stats, version 1.21, RRID:SCR_002105), QualiMap (version 2.3, RRID:SCR_001209) and MultiQC (version 1.7, RRID:SCR_014982). Samples with <70% of unique reads mapped, <750 thousand unique reads, or a GC content <40% were excluded from further analysis. Reads were counted per gene with subread (featureCounts, version 2.0.6, RRID:SCR_009803). Mapping and quality assessment for the Lund samples was performed as previously described^62^.

The ComBat_seq function from the sva package^68^ was used to correct for batch effects (NKI and Oslo vs Lund). The TMM function from the edgeR package^69^ was used to correct for library size, after which counts were converted to counts per million using the cpm function from edgeR^69^.

#### Normal breast tissue RNA data

GTEx normal breast mammary tissue gene counts data (v10)^30^ were corrected for collection site batch effects using SMCENTER annotation and TMM-normalized based on the method described above.

### Patient classifiers

#### PAM50

PAM50 classification^70^ was calculated using DCIS expression data (*n* = 97) according to the subgroup centering methods^71^ which adjusts for differences in ER and HER2 positivity frequency between this DCIS dataset and the invasive breast cancer dataset the PAM50 was defined on.

#### Oncotype DX

The Oncotype DX risk scores were calculated using DCIS expression data (*n* = 97) according to the methods described by Solin et al. 2013^35^. However, the original methods are based on RT-qPCR, so additional normalization steps were required as described above.

### Genomic prognosticator for ER + HER2- samples

ER + HER2- DCIS patients (*n* = 37) were classified using WGS-based IBC prognosticator^11^ into high-risk, low-mid risk and low-risk categories. To be classified as high-risk, patients had either *TP53* driver mutation or were *TP53*-wildtype with SV count > 90 and SBS2/13 exposure > 25%.

#### SHORE expression assessment using permutation test

To compare the expression of genes within SHOREs with expression of genes in the rest of the genome (non-SHORE genes), we performed a permutation test. We used normalized RNA-seq data for DCIS and normal breast tissue, respectively. For each sample, 10,000 random sets of non-SHORE genes were selected, matching the total SHORE gene count. For DCIS analysis, if any SHORE genes were identified as amplification drivers, or were located on an amplicon according to AmpliconClassifier predictions or within a SHORE overlapping with an amplicon, they were excluded from the analysis, and the size of the random gene sets was adjusted to match the remaining SHORE gene count for the given sample.

For each of the 10,000 non-SHORE gene sets, we calculated the mean expression *y*_*i*_ (*i* = 1…10,000) across all samples. We then calculated *z*, the mean expression of all SHORE genes across all samples and derived a p-value as:

2 $$p=\frac{\mathop{\sum }_{i}{{{\rm{S}}}}\left[{y}_{i} > z\right]+1}{10001}$$

where *S* is the number of permutations in which the condition *y*_*i*_ > *z* is satisfied.

In the per-sample permutation analysis performed for DCIS, *y*_*i*_ was calculated for each sample individually, and *z* was mean expression of SHORE gene in that sample. The p-value was calculated following the same formula and adjusted for multiple testing using Benjamini-Hochberg False Discovery Rate correction^72^. The z-score was calculated as the difference between the observed mean and the mean of the sample distribution, divided by the standard deviation of the sample distribution.

#### Actionability

To identify variants that are clinically actionable in BC, we searched against the FDA-recognized biomarkers variants (Level of evidence 1) available on the OncoKB database (https://www.oncokb.org/) as of 24^th^ of May, 2025^73^.

### Reporting summary

Further information on research design is available in the Nature Portfolio Reporting Summary linked to this article.

## Supplementary information

Supplementary Information

Description of Additional Supplementary Files

Supplementary Data 1

Supplementary Data 2

Supplementary Data 3

Supplementary Data 4

Supplementary Data 5

Supplementary Data 6

Supplementary Data 7

Supplementary Data 8

Reporting Summary

Transparent Peer Review file

## Source data

Source Data

## Data Availability

DCIS RNA-sequencing-based gene expression data from the Swedish cohort (Lund) have been published previously and are available at Mendeley Data as a publicly accessible dataset [10.17632/yzxtxn4nmd.1]^62^. RNA sequencing data for the 77 samples generated in this study (Oslo and NKI cohorts) have been deposited in European-Genome Phenome Archive (EGA), accession number EGAD50000002123. Raw DCIS WGS data have been deposited in EGA for a total of 77 tumor/normal pairs, accession numbers EGAD50000002071 (51 tumor/normal pairs (NKI cohort), https://ega-archive.org/datasets/EGAD50000002071) and EGAD50000002237 (26 tumor/normal pairs from the Oslo cohort, with restrictions for use for somatic mutation calling only due to specific restrictions imposed by the ethical approval at sample collection, https://ega-archive.org/datasets/EGAD50000002237). Access to all aforementioned DCIS datasets, for academic use only, is subjected to completion of a Data Access Agreement and is granted on a project-specific basis that complies with the terms and conditions of the data access agreement. Estimated response time to data access requests is 6–8 weeks. Project-specific duration of the of data access will be specified as part of the data access agreement. The raw whole genome sequencing data for the remaining 36 samples in this study from the Lund cohort, which were collected as part of the SCANB study, are not publicly available due to patient privacy requirements under Swedish law and specific patient consent. Requests for access can be made depending on the request’s alignment with Swedish data privacy laws, ethical permissions, and specific informed patient consent, defined through a formal data request application. Data requests should be made to the SCAN-B Steering Group, using the SCAN-B research project application template form and contact address [scanb@med.lu.se] listed on the SCAN-B website [https://www.scan-b.lu.se/en/scientists]. Processing time of initial requests is estimated at 6–8 weeks depending on the scheduled steering group meetings. Depending on the nature of a request and the geographic location of the applicant/host university, additional data transfer agreements may be required as determined by data protection officers at Lund University, Sweden, to assure that any relevant and current legal restrictions imposed by Swedish law and the European Union concerning research data sharing are followed. Filtered somatic mutation calls for DCIS samples are available in Mendeley data [10.17632/h8fv2tc8d4.1]. Mutational profiles and signatures of all DCIS samples can be viewed on SIGNAL website [https://signal.mutationalsignatures.com/explore/main/cancer/signatures?mutationType=1&study=13]. ICGC data used in this study were published previously and deposited in EGA under accession codes EGAS00001001178 and EGAD00001002740. The Genomics England data used in this analysis are available in the supplementary data for Black et al.^11^. Data can also be accessed from the Genomics England in the Research Environment subject to a collaborative agreement that adheres to patient led governance. For more information, access the relevant information on the Genomics England website: [https://www.genomicsengland.co.uk/research]. Hartwig Medical Foundation Data can be accessed at [www.hartwigmedicalfoundation.nl/en]. RS1 hotspot regions are publicly available in the supplementary table 1 from^27^. The publicly accessible origin of replication regions can be obtained from the supplementary table 1 in^32^. GTEx normal breast mammary tissue gene counts data (v10) are publicly available at the GTEx Portal [https://www.gtexportal.org/home/downloads/adult-gtex/]^30^. R-loop regions are publicly accessible at R-loopBase database [https://rloopbase.nju.edu.cn/]^66^. BrdU peaks data are available in the Gene Expression Omnibus database under accession code GSE267038^34^. Other data generated in this study are available within the article and its supplementary data files. Source data are provided with this paper.
