# Supplementary material for: High-depth whole genome sequencing of premalignant breast lesions reveals rearrangement hotspots and personalized management opportunities
Source: Nat Commun. 2026 May 19;17:6931. doi: 10.1038/s41467-026-72952-1 (PMC13389004; doi:10.1038/s41467-026-72952-1)
Supplement: Supplementary file 1 — Supplementary Information [file 41467_2026_72952_MOESM1_ESM.pdf]

## Supplementary Figures S1 - 7.

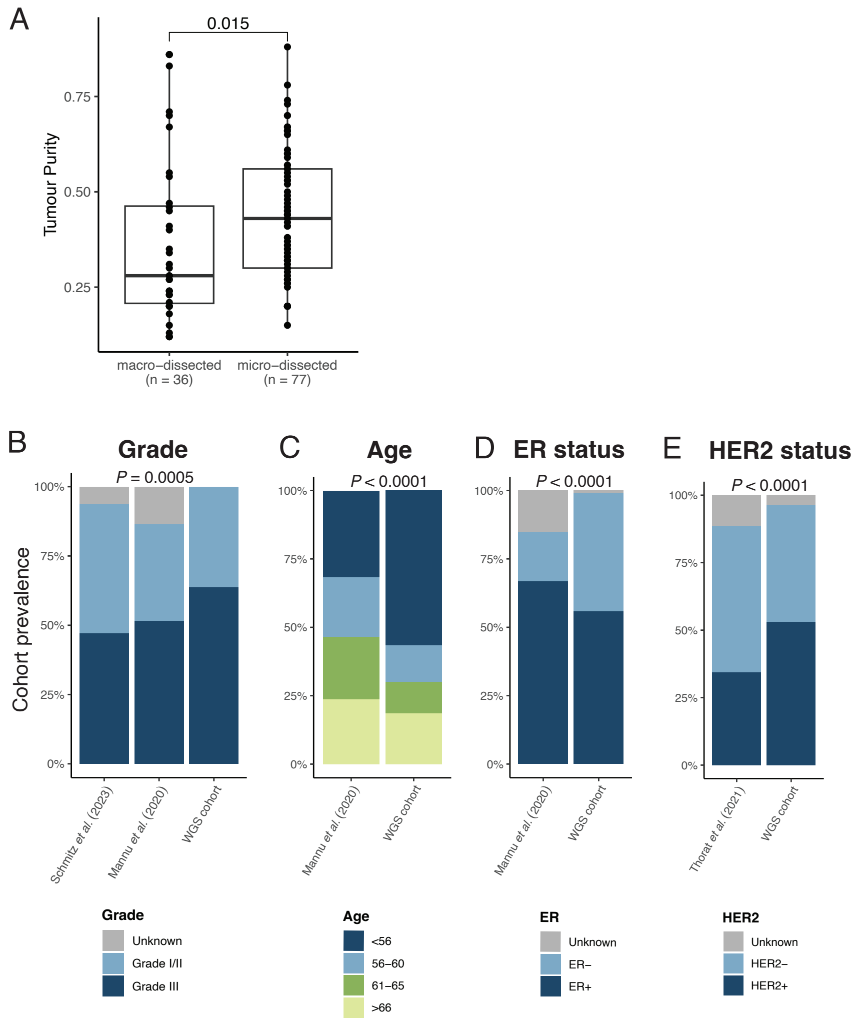


**Supplementary Figure S1. Cohort summary and comparison to published DCIS cohorts**. (**A**) Comparison of tumor purity between macro- and micro-dissected samples ($n$ = 113). Wilcoxon rank-sum two-sided test. Boxplots show the median, 25th–75th percentiles, with whiskers extending to ±1.5 × IQR (the interquartile range). (**B – E**) Comparisons of WGS DCIS samples ($n$ = 113) with population-based datasets: Schmitz *et al.* (2023) ($n$ = 47,695 samples) (1) and Mannu *et al.* (2020) ($n$ = 35,024 samples) (2) for (**B**) Grade (**C**) Age, and (**D**) ER status. (**E**) HER2 status was compared to the UK/ANZ DCIS randomized trial: Thorat *et al.* (2021) (3) ($n$ = 713 samples). Fisher's exact two-sided test. Source data are provided as a Source Data file.

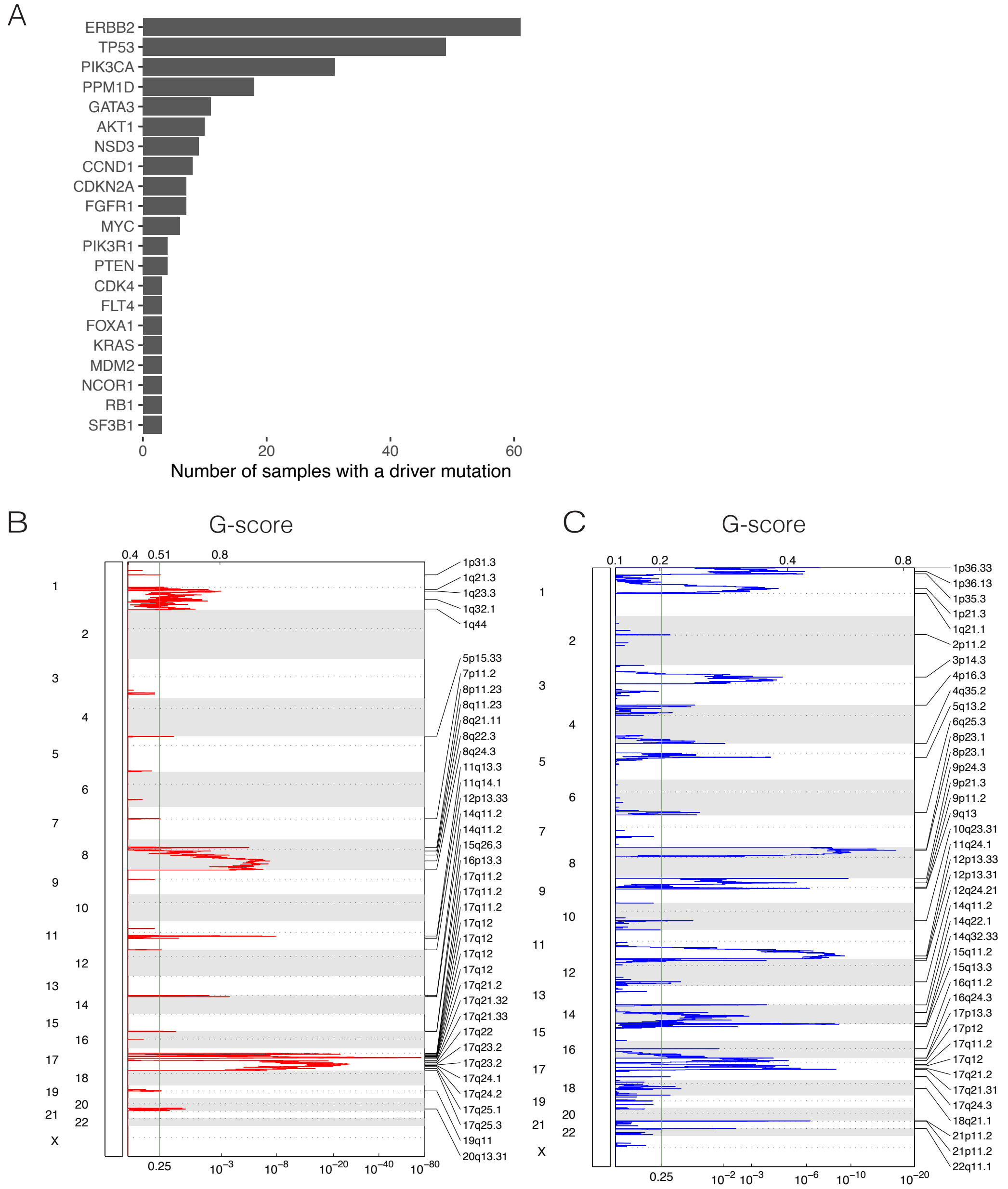


**Supplementary Figure S2. Driver and copy number aberration (CNA) summary**. (**A**) Distribution of driver mutations in DCIS samples ($n$ = 113). (**B – C**) Cohort-wide significantly amplified (**B**) and deleted (**C**) chromosomal regions as identified by GISTIC2.0 ($q$ = 0.25). Source data are provided as a Source Data file.

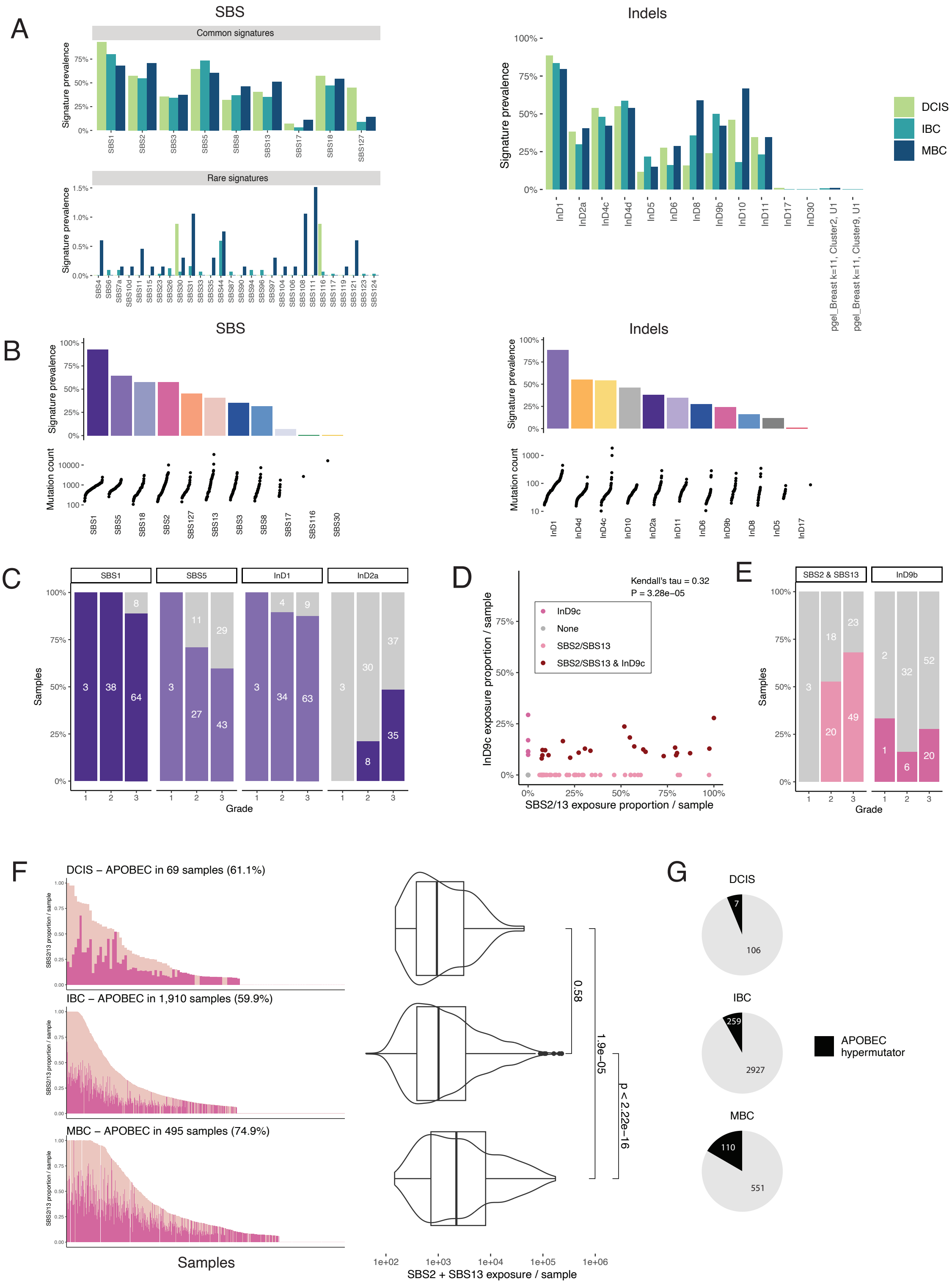


**Supplementary Figure S3. Mutational signatures across BC stages**. (**A**) SBS and indel signature prevalence – fraction of samples assigned the signature by BC stage: DCIS (*n* = 113 samples), IBC (*n* = 3,186 samples) and MBC (*n* = 661 samples) (4–6). (**B**) SBS (left) and indel (right) mutational signatures in DCIS samples (*n* = 113). Top panel, fraction of samples assigned the signature; bottom panel, mutation count of signature exposures. (**C**) Prevalence of endogenous signatures SBS1, SBS5, InD1 and InD2a grouped by grade in DCIS samples (*n* =

113). (**D**) Association between APOBEC SBS2/SBS13 and InD9b signatures in DCIS samples (*n* = 113). Kendall correlation. (**E**) Prevalence of APOBEC SBS and indel signatures grouped by grade in DCIS samples (*n* = 113). (**F**) Prevalence (right) and burden (left) of APOBEC SBS2/SBS13 across BC stages (4–6). Burden comparison using Wilcoxon rank-sum two-sided test. The boxplots show the median, 25th–75th percentiles, with whiskers extending to ±1.5 × IQR (the interquartile range). (**G**) Increasing prevalence of APOBEC hypermutator samples (SBS2/SBS13 sample exposure > 80%) across BC cohorts. Source data are provided as a Source Data file.

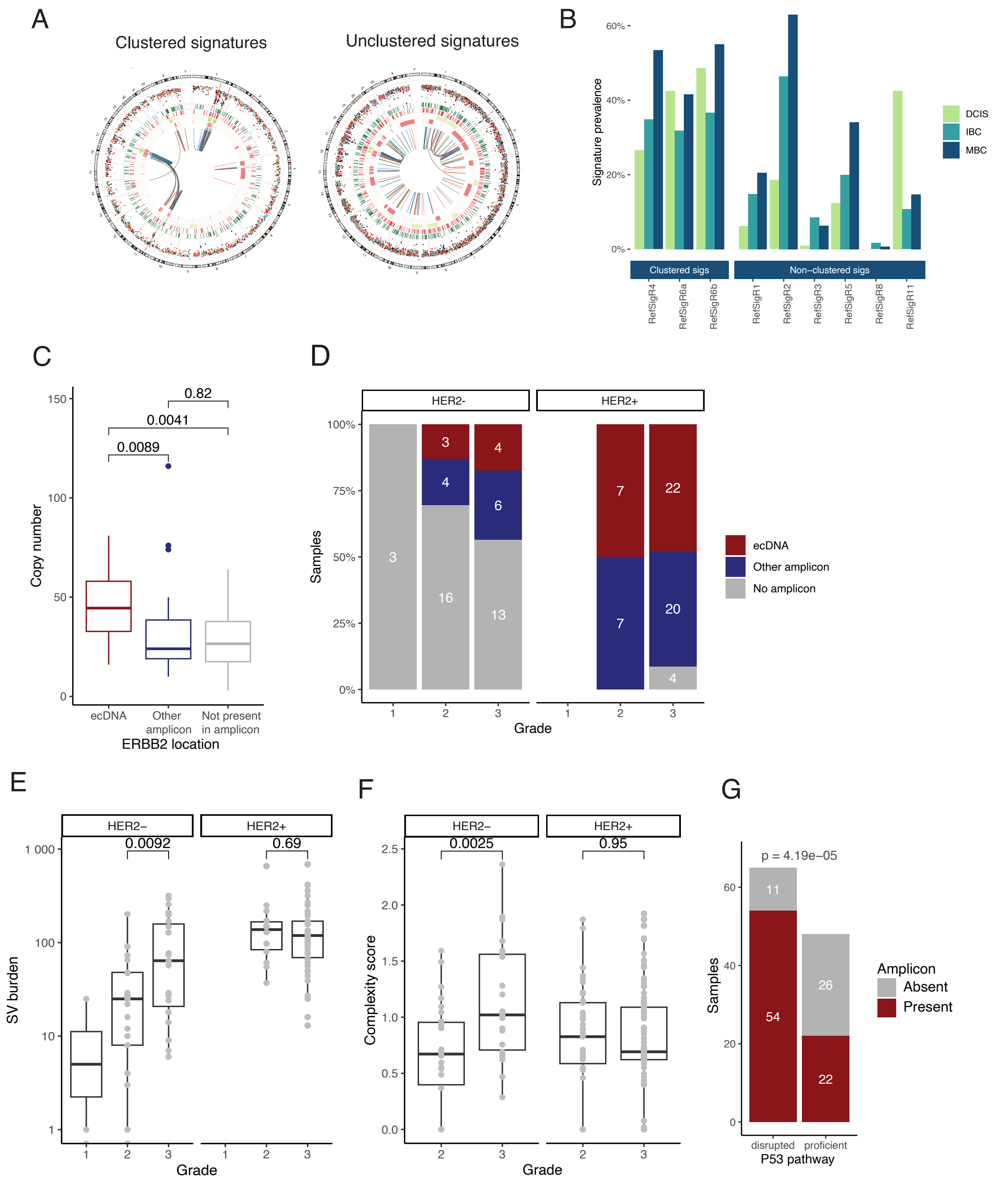


**Supplementary Figure S4. Impact of clustered SVs and amplicons in DCIS**. (**A**) Examples of genome plots with prevalent clustered SVs (left) and unclustered SVs (right). Features depicted in circos plots from outermost rings heading inwards are as described in Davies et al. (2017): Karyotypic ideogram outermost. Base substitutions next, plotted as rainfall plots (log10 intermutation distance on radial axis, dot colors: blue, C>A; black, C>G; red, C>T; grey, T>A; green, T>C; pink, T>G). Ring with short green lines, insertions; ring with short red lines, deletions. Major copy number allele ring (green, gain), minor copy number allele ring (red, loss). Central lines represent rearrangements (green, tandem duplications; red, deletions; blue, inversions; black, translocations). (**B**) RS prevalence by BC stage. DCIS (*n* = 113 samples),

IBC ($n$ = 3,186 samples) and MBC ($n$ = 661 samples) (4–6). (**C**) WGS-derived *ERBB2* CN by the amplicon type in HER2-positive samples ($n$ = 60). Two-sided $t$-test. (**D**) Distribution and prevalence of ecDNA and amplicon presence by HER2 status and grade ($n$ = 109 samples). Samples with unknown HER2 status ($n$ = 4) were omitted. (**E**) Comparison of SV burden in DCIS samples ($n$ = 109) by HER2 status and grade. Samples with unknown HER2 status ($n$ = 4) were omitted. Wilcoxon rank-sum two-sided test. (**F**) Comparison of complexity score of amplicons ($n$ = 196) from AmpliconSuite pipeline by HER2 status and grade. Samples with unknown HER2 status ($n$ = 4) were omitted. Two-sided $t$-test. (**G**) Association between presence of amplicon and the disruption of p53 pathway, including mutations in *TP53*, *PPM1D*, *MDM2*, and *MDM4*. Fisher's exact two-sided test ($n$ = 113 samples). Boxplots (C,E,F) show the median, 25th–75th percentiles, with whiskers extending to ±1.5 × IQR (the interquartile range). Source data are provided as a Source Data file.

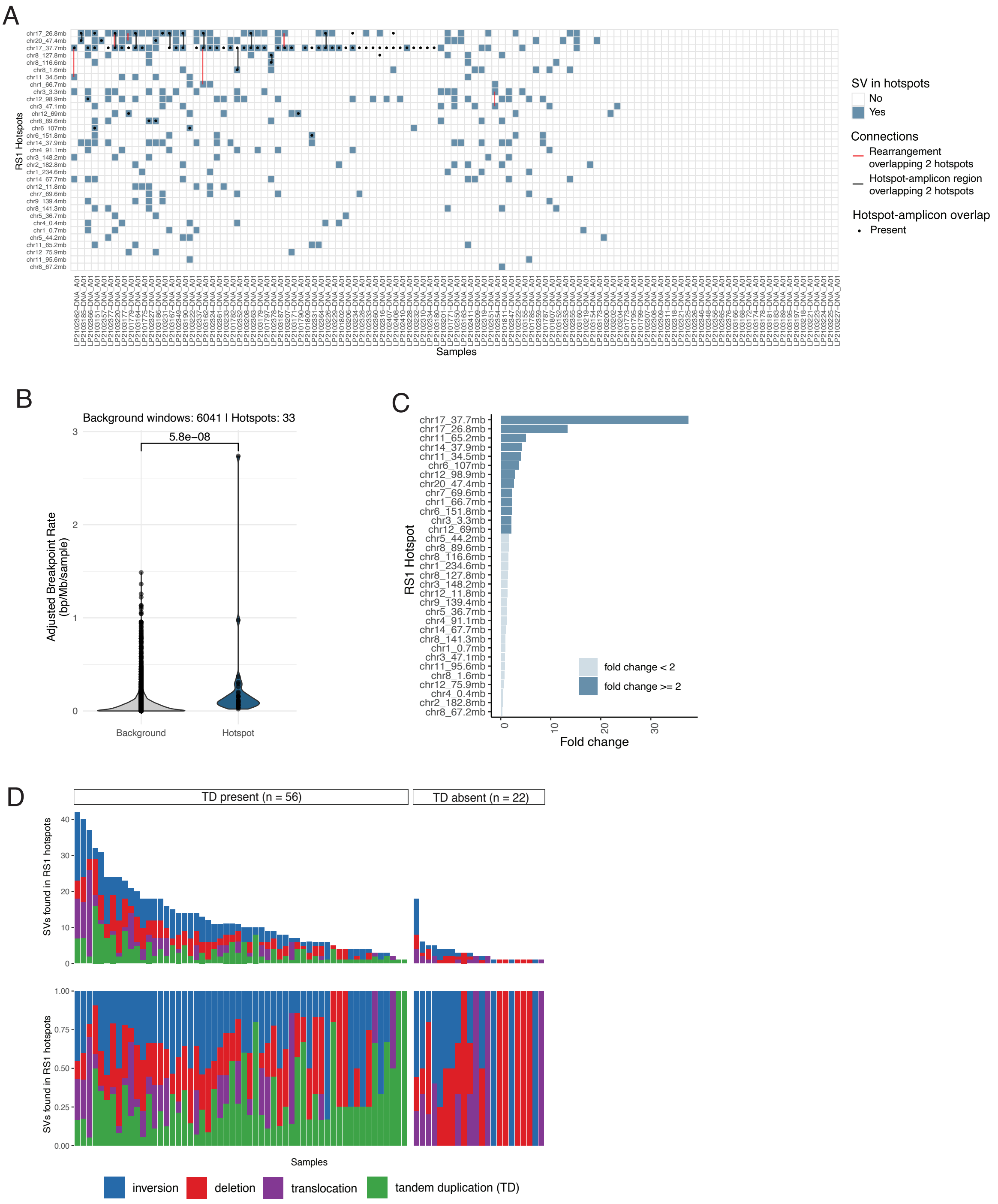


**Supplementary Figure S5. RS1 hotspot characteristics.** (**A**) Sample overview of RS1 hotspots by the presence of SV, hotspot-amplicon overlap, rearrangements overlapping 2 hotspots (S2-SVs), and hotspots-amplicons regions overlapping 2 hotspots. (**B**) The adjusted breakpoint rate in the background genome divided into the 500kb windows compared to the RS1 hotspots regions across DCIS samples ($n$ = 113). Wilcoxon rank-sum two-sided test. (**C**) Fold change in the breakpoint rate of the RS1 hotspots compared to the average background breakpoint rate (0.07 bp/Mb/sample). (**D**) SV composition within RS1 hotspots by sample ($n$

= 78), stratified by the presence of tandem duplication (TD) in the hotspots. Absolute SV burden (top), proportion (bottom). Source data are provided as a Source Data file.

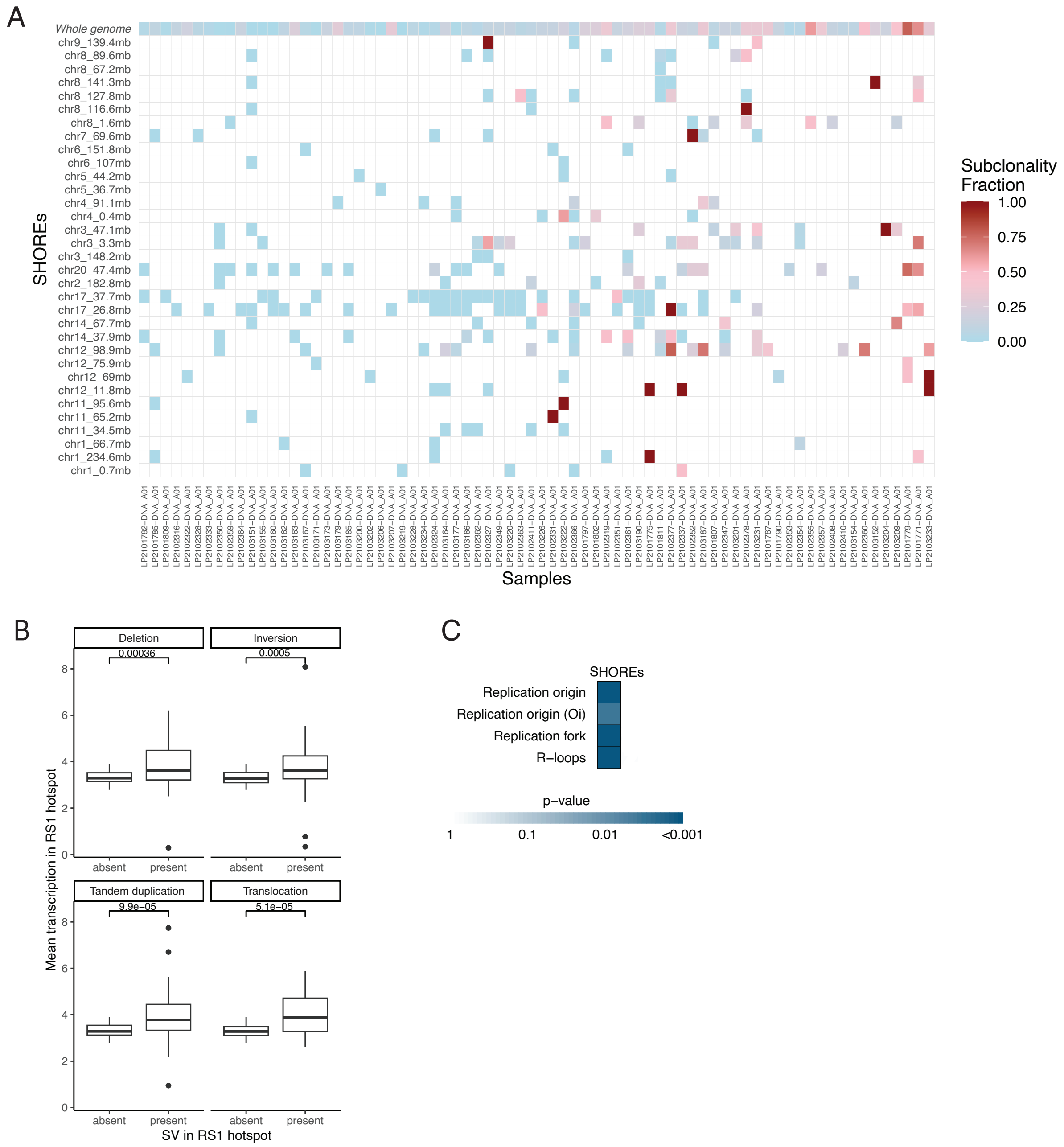


**Supplementary Figure S6. Additional SHORE characteristics in DCIS.** (**A**) Summary of subclonality within SHOREs with a SV present (*n* = 250 regions). The samples were ordered by the number of clonal SHOREs per sample and the number of largely subclonal SHOREs. The SHOREs were ordered by a proportion of clonal SHOREs. (**B**) Paired comparison of mean transcription rate in SHOREs harboring SV versus in SHOREs without SV in the same sample grouped by the SV type (*n* = 97 samples). Wilcoxon paired signed-rank two-sided test. The boxplots show the median, 25th–75th percentiles, with whiskers extending to ±1.5 × IQR (the interquartile range). (**C**) Enrichment of the genomic features (7–9) in the SHORE regions (*n* = 33). Empirical p-values were estimated using Monte Carlo simulations. Source data are provided as a Source Data file.

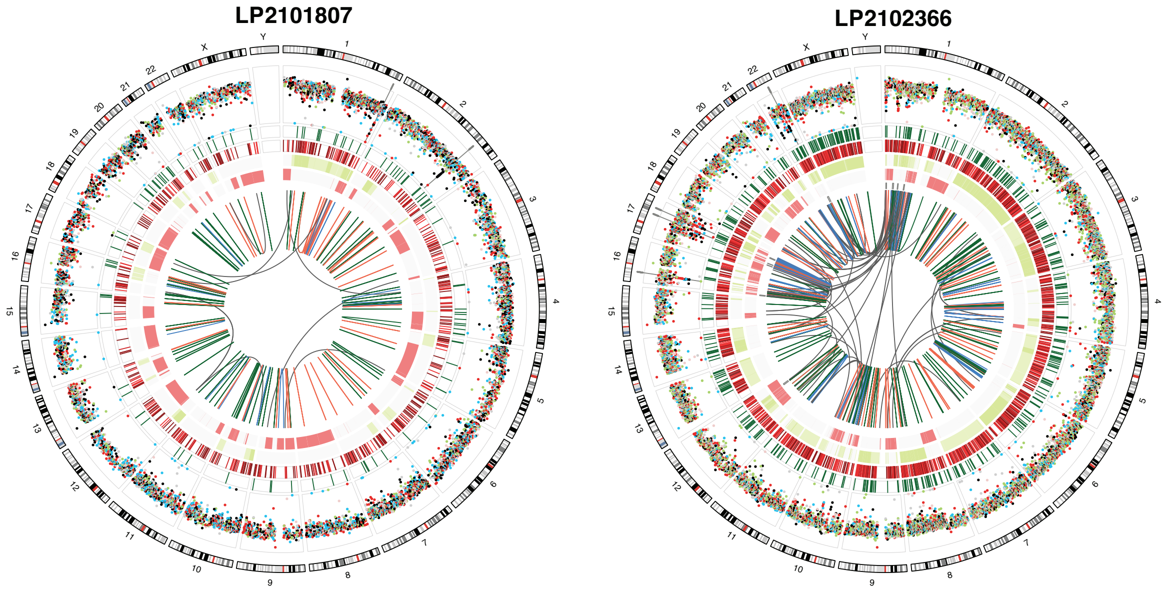


**Supplementary Figure S7. Genome plots of HRDetect-high samples (*n* = 2)**. Left – LP2101807 sample – HRDetect score: 0.99. Right – LP2102366 sample, HRDetect score: 0.94. Genome plots features are as described in Supplementary Fig. S4A. Source data are provided as a Source Data file.

***Supplementary reference list***


1. Schmitz RSJM, Belt-Dusebout AW van den, Clements K, Ren Y, Cresta C, Timbres J, et al. Association of DCIS size and margin status with risk of developing breast cancer post-treatment: multinational, pooled cohort study. BMJ. 2023 Oct 30;383:e076022.

2. Mannu GS, Wang Z, Broggio J, Charman J, Cheung S, Kearins O, et al. Invasive breast cancer and breast cancer mortality after ductal carcinoma in situ in women attending for breast screening in England, 1988-2014: population based observational cohort study. BMJ. 2020 May 27;369:m1570.

3. Thorat MA, Levey PM, Jones JL, Pinder SE, Bundred NJ, Fentiman IS, et al. Prognostic and Predictive Value of HER2 Expression in Ductal Carcinoma In Situ: Results from the UK/ANZ DCIS Randomized Trial. Clinical Cancer Research. 2021 Oct 1;27(19):5317–24.

4. Nik-Zainal S, Davies H, Staaf J, Ramakrishna M, Glodzik D, Zou X, et al. Landscape of somatic mutations in 560 breast cancer whole-genome sequences. Nature. 2016 Jun;534(7605):47–54.

5. Black D, Davies HR, Koh GCC, Chmelova L, Cubric M, Chan GC, et al. Clinical potential of whole-genome data linked to mortality statistics in patients with breast cancer in the UK: a retrospective analysis. The Lancet Oncology. 2025 Nov 1;26(11):1417–31.

6. Angus L, Smid M, Wilting SM, van Riet J, Van Hoeck A, Nguyen L, et al. The genomic landscape of metastatic breast cancer highlights changes in mutation and signature frequencies. Nat Genet. 2019 Oct;51(10):1450–8.

7. Macheret M, Halazonetis TD. Intragenic origins due to short G1 phases underlie oncogene-induced DNA replication stress. Nature. 2018 Mar;555(7694):112–6.

8. Lin R, Zhong X, Zhou Y, Geng H, Hu Q, Huang Z, et al. R-loopBase: a knowledgebase for genome-wide R-loop formation and regulation. Nucleic Acids Res. 2022 Jan 7;50(D1):D303–15.

9. Rojas P, Wang J, Guglielmi G, Sadurnì MM, Pavlou L, Leung GHD, et al. Genome-wide identification of replication fork stalling/pausing sites and the interplay between RNA Pol II transcription and DNA replication progression. Genome Biology. 2024 May 21;25:126.
