# Supplementary material for: High-depth whole genome sequencing of premalignant breast lesions reveals rearrangement hotspots and personalized management opportunities
Source: Nat Commun. 2026 May 19;17:6931. doi: 10.1038/s41467-026-72952-1 (PMC13389004; doi:10.1038/s41467-026-72952-1)
Supplement: Supplementary file 2 — Description of Additional Supplementary Files [file 41467_2026_72952_MOESM2_ESM.pdf]

## Description of Additional Supplementary Information

Title: Supplementary Data 1
Description: Overview of clinical and genomic information for DCIS samples (n = 113).

Title: Supplementary Data 2
Description: List of somatic mutational drivers in DCIS samples (n = 113).

Title: Supplementary Data 3
Description: Single-base substitution (SBS), rearrangement (RS), and indel (InD) mutational signature exposures for DCIS samples (n = 113).

Title: Supplementary Data 4
Description: Summary of amplicon characteristics identified in DCIS samples (n = 113) provided from AmpliconSuite outputs.

Title: Supplementary Data 5
Description: Copy number of ERBB2 gene in DCIS samples (n = 113).

Title: Supplementary Data 6
Description: Overlap between RS1 hotspots and amplicons identified in DCIS samples (n = 113).

Title: Supplementary Data 7
Description: Overview of structural variants in DCIS samples (n = 113) located within SHORE regions. S2-SVs highlight cases where both breakpoints are located within SHOREs.

Title: Supplementary Data 8
Description: Count of genomic features and mean expression of normal breast tissue found within SHOREs and randomly resampled SHORE-like regions.
