# Supplementary material for: High-depth whole genome sequencing of premalignant breast lesions reveals rearrangement hotspots and personalized management opportunities
Source: Nat Commun. 2026 May 19;17:6931. doi: 10.1038/s41467-026-72952-1 (PMC13389004; doi:10.1038/s41467-026-72952-1)
Supplement: Supplementary file 11 — Reporting Summary [file 41467_2026_72952_MOESM11_ESM.pdf]

nature portfolio

Corresponding author(s): Serena Nik-Zainal

Last updated by author(s): 2026 04 27

Reporting Summary

Nature Portfolio wishes to improve the reproducibility of the work that we publish. This form provides structure for consistency and transparency in reporting. For further information on Nature Portfolio policies, see our Editorial Policies and the Editorial Policy Checklist.

Statistics

For all statistical analyses, confirm that the following items are present in the figure legend, table legend, main text, or Methods section.

n/a	Confirmed	
☐	☒	The exact sample size (*n*) for each experimental group/condition, given as a discrete number and unit of measurement
☐	☒	A statement on whether measurements were taken from distinct samples or whether the same sample was measured repeatedly
☐	☒	The statistical test(s) used AND whether they are one- or two-sided<br>*Only common tests should be described solely by name; describe more complex techniques in the Methods section.*
☐	☒	A description of all covariates tested
☐	☒	A description of any assumptions or corrections, such as tests of normality and adjustment for multiple comparisons
☐	☒	A full description of the statistical parameters including central tendency (e.g. means) or other basic estimates (e.g. regression coefficient) AND variation (e.g. standard deviation) or associated estimates of uncertainty (e.g. confidence intervals)
☐	☒	For null hypothesis testing, the test statistic (e.g. *F*, *t*, *r*) with confidence intervals, effect sizes, degrees of freedom and *P* value noted<br>*Give P values as exact values whenever suitable.*
☒	☐	For Bayesian analysis, information on the choice of priors and Markov chain Monte Carlo settings
☒	☐	For hierarchical and complex designs, identification of the appropriate level for tests and full reporting of outcomes
☒	☐	Estimates of effect sizes (e.g. Cohen's *d*, Pearson's *r*), indicating how they were calculated

Our web collection on statistics for biologists contains articles on many of the points above.

Software and code

Policy information about availability of computer code

Data collection	No software was used for data collection
Data analysis	Variant calling: CaVEMan (v.1.13.15), Pindel (v3.2.0), ASCAT (v4.2.1), BRASS (v6.2.1), (contained in dockstore-cgpwgs v2.1.1 available at https://quay.io/repository/wtsicgp/dockstore-cgpwgs), GISTIC 2 v.2.0.23, mapping: bwa mem (v.0.7.17-r1188), mutational signature analysis; signature.tools.lib(v2.4.4), . Amplicon predictions: AmpliconArchitect (v1.3.r5), CNVKit (v0.9.10), AmpliconClassifier (v1.2.1), topography.tools.lib (v1.0.0). RNAsequencing: cutadapt (version 4.9, RRID:SCR_011841), reads were aligned to the human genome (Gencode release 47 (62)) with STAR (version 2.7.11d, RRID:SCR_004463), FastQC (version 0.12.1, RRID:SCR_014583), Picard (CollectRnaSeqMetrics, version 3.2.0, RRID:SCR_006525), Samtools (stats, version 1.21, RRID:SCR_002105), QualiMap (version 2.3, RRID:SCR_001209), MultiQC (version 1.7, RRID:SCR_014982), featureCounts, version 2.0.6, RRID:SCR_009803. MutationTimeR algorithm (v1.00.2).

For manuscripts utilizing custom algorithms or software that are central to the research but not yet described in published literature, software must be made available to editors and reviewers. We strongly encourage code deposition in a community repository (e.g. GitHub). See the Nature Portfolio guidelines for submitting code & software for further information.

Data

Policy information about availability of data

All manuscripts must include a data availability statement. This statement should provide the following information, where applicable:

- Accession codes, unique identifiers, or web links for publicly available datasets
- A description of any restrictions on data availability
- For clinical datasets or third party data, please ensure that the statement adheres to our policy

DCIS RNA-sequencing-based gene expression data from the Swedish cohort (Lund) have been published previously and are available at Mendeley Data as a publicly accessible dataset [https://doi.org/10.17632/yzxtxn4nmd.1] ((Staaf et al. NPJ Breast Cancer. 2022 Aug 16;8:94.). RNA sequencing data for the 77 samples generated in this study (Oslo and NKI cohorts) have been deposited in European-Genome Phenome Archive (EGA), accession number EGAD50000002123 [https://ega-archive.org/datasets/EGAD50000002123]. Raw DCIS WGS data have been deposited in EGA for a total of 77 tumour/normal pairs, accession numbers EGAD50000002071 (51 tumour/normal pairs (NKI cohort), https://ega-archive.org/datasets/EGAD50000002071) and EGAD50000002237 (26 tumour/normal pairs from the Oslo cohort, with restrictions for use for somatic mutation calling only due to specific restrictions imposed by the ethical approval at sample collection, https://ega-archive.org/datasets/EGAD50000002237). Access to all aforementioned DCIS datasets, for academic use only, is subjected to completion of a Data Access Agreement and is granted on a project-specific basis that complies with the terms and conditions of the data access agreement. Estimated response time to data access requests is 6-8 weeks. Project-specific duration of the of data access will be specified as part of the data access agreement. The raw whole genome sequencing data for the remaining 36 samples in this study from the Lund cohort, which were collected as part of the SCANB study, are not publicly available due to patient privacy requirements under Swedish law and specific patient consent. Requests for access can be made depending on the request's alignment with Swedish data privacy laws, ethical permissions, and specific informed patient consent, defined through a formal data request application. Data requests should be made to the SCAN-B Steering Group, using the SCAN-B research project application template form and contact address [scanb@med.lu.se] listed on the SCAN-B website [https://www.scan-b.lu.se/en/scientists]. Processing time of initial requests is estimated at 6–8 weeks depending on the scheduled steering group meetings. Depending on the nature of a request and the geographic location of the applicant/host university, additional data transfer agreements may be required as determined by data protection officers at Lund University, Sweden, to assure that any relevant and current legal restrictions imposed by Swedish law and the European Union concerning research data sharing are followed.Filtered somatic mutation calls for DCIS samples are available in Mendeley data [https://doi.org/10.17632/h8fv2tc8d4.1]. Mutational profiles and signatures of all DCIS samples can be viewed on SIGNAL website [https://signal.mutationalsignatures.com/explore/main/cancer/signatures?mutationType=1&study=13].

ICGC data used in this study were published previously and deposited in EGA under accession codes EGAS00001001178 [https://ega-archive.org/studies/EGAS00001001178] and EGAD00001002740 [https://ega-archive.org/datasets/EGAD00001002740]. The Genomics England data used in this analysis are available in the supplementary data for Black et al. (Lancet Oncol. 2025 Nov 1;26(11):1417–31). Data can also be accessed from the Genomics England in the Research Environment subject to a collaborative agreement that adheres to patient led governance. For more information, access the relevant information on the Genomics England website: [https://www.genomicsengland.co.uk/research]. Hartwig Medical Foundation Data can be accessed at [www.hartwigmedicalfoundation.nl/en]. RS1 hotspot regions are publicly available in the supplementary table 1 from (27). The publicly accessible origin of replication regions can be obtained from the supplementary table 1 in (Glodzik et al Nat Genet. 2017 Mar;49(3):341–8). GTEx normal breast mammary tissue gene counts data (v10) are publicly available at the GTEx Portal [https://www.gtexportal.org/home/downloads/adult-gtex/] (The GTEx Consortium. Science. 2020 Sep 11;369(6509):1318–30). R-loop regions are publicly accessible at R-loopBase database [https://rloopbase.nju.edu.cn/] (Lin et al. Nucleic Acids Res. 2022 Jan 7;50(D1):D303–15.). BrdU peaks data are available in the Gene Expression Omnibus database under accession code GSE267038 [https://www.ncbi.nlm.nih.gov/geo/query/acc.cgi?acc=GSE267038] (Rojas et al. Genome Biol. 2024 May 21;25:126.). Other data generated in this study are available within the article and its supplementary data files. Source data are provided with this paper.

Research involving human participants, their data, or biological material

Policy information about studies with human participants or human data. See also policy information about sex, gender (identity/presentation), and sexual orientation and race, ethnicity and racism.

Reporting on sex and gender	Sex and gender were not considered in the study design
Reporting on race, ethnicity, or other socially relevant groupings	Race, ethnicity snd other socially relevant groups were not considered in the study design
Population characteristics	The patients included in this study were diagnosed with Ductal Carcinoma In Stu (DCIS). Age groups are included in supplementary table 1
Recruitment	113 patients diagnosed with DCIS were recruited via three 3 collection studies: Oslo University Hospital, Norway; Netherland Cancer Institute (NKI), Netherlands; Lund University, Sweden. Oslo samples were collected as part of the Oslo2 breast cancer observational study. The Lund cohort were enrolled in the SCAN-B study (NCT02306096) during 2010-2014. The NKI sample cohort was collected from DCIS patients treated at the Netherlands Cancer Institute between 2017 and 2022. As described in the text, samples were biased towards more aggressive higher grade DCIS due to limited available material for DNA extraction from low-grade lesions which tend to be smaller in size.
Ethics oversight	The use of the samples was subject to patient consent and ethical approval of the respective collection centre. The Oslo2 breast cancer observational study had ethics approval numbers 2016/433 and 2019/657. Ethical approval was given for the SCAN-B study (Registration numbers 2009/658, 2010/383, 2012/58, 2013/459, 2014/521, 2015/277, 2016/541, 2016/742, 2016/944, 2018/267, 2019/01252, and 2024-02040-02) by the Regional Ethical Review Board in Lund, Sweden, governed by the Swedish Ethical Review Authority, Box 2110, 750 02 Uppsala, Sweden. The NKI study was approved by the Institutional Review Board at the NKI under approval number CFMPB658.

Note that full information on the approval of the study protocol must also be provided in the manuscript.

Field-specific reporting

Please select the one below that is the best fit for your research. If you are not sure, read the appropriate sections before making your selection.

- [x] Life sciences
- [ ] Behavioural & social sciences
- [ ] Ecological, evolutionary & environmental sciences

For a reference copy of the document with all sections, see nature.com/documents/nr-reporting-summary-flat.pdf

Life sciences study design

All studies must disclose on these points even when the disclosure is negative.

Sample size	No samples size calculation was performed. The all available samples were used
Data exclusions	No data was excluded. Whole genome sequencing data was subject to standard QC checks prior to inclusion in the study
Replication	A suitable DCIS data set was not available for replication. Results were compared and contrasted to 2 other large breast whole genome cohorts. Control of covariants was not applicable to this study. A description of the cohort is included in the text with comparison to other published DCIS cohorts in the text and supplementary figure S1.
Randomization	Randomization was not required as part of this study
Blinding	Blinding was not applicable to this study as it is a description based study and does not include outcome data .

Reporting for specific materials, systems and methods

We require information from authors about some types of materials, experimental systems and methods used in many studies. Here, indicate whether each material, system or method listed is relevant to your study. If you are not sure if a list item applies to your research, read the appropriate section before selecting a response.

Materials & experimental systems

n/a	Involved in the study	
[ ]	[x]	Antibodies
[x]	[ ]	Eukaryotic cell lines
[x]	[ ]	Palaeontology and archaeology
[x]	[ ]	Animals and other organisms
[ ]	[x]	Clinical data
[x]	[ ]	Dual use research of concern
[x]	[ ]	Plants

Methods

n/a	Involved in the study	
[x]	[ ]	ChIP-seq
[x]	[ ]	Flow cytometry
[x]	[ ]	MRI-based neuroimaging

Antibodies

Antibodies used	VENTANA® anti-HER2/neu (4B5) Rabbit Monoclonal Primary Antibody (Roche), CONFIRM anti-Estrogen Receptor (ER) (SP1) Rabbit Monoclonal Primary Antibody (Roche).
Validation	Details can be found on the manufacturer's website:<br>https://diagnostics.roche.com/global/en/products/lab/her2-neu-4b5-ventana-rtd001197.html<br>https://diagnostics.roche.com/se/en/products/lab/estrogen-receptor-sp1-confirm-pid00000060.html

Clinical data

Policy information about clinical studies

All manuscripts should comply with the ICMJE guidelines for publication of clinical research and a completed CONSORT checklist must be included with all submissions.

Clinical trial registration	This study was not part of a clinical trial
Study protocol	*Note where the full trial protocol can be accessed OR if not available, explain why.*
Data collection	*Describe the settings and locales of data collection, noting the time periods of recruitment and data collection.*
Outcomes	*Describe how you pre-defined primary and secondary outcome measures and how you assessed these measures.*

Plants

Seed stocks

Report on the source of all seed stocks or other plant material used. If applicable, state the seed stock centre and catalogue number. If plant specimens were collected from the field, describe the collection location, date and sampling procedures.

Novel plant genotypes

Describe the methods by which all novel plant genotypes were produced. This includes those generated by transgenic approaches, gene editing, chemical/radiation-based mutagenesis and hybridization. For transgenic lines, describe the transformation method, the number of independent lines analyzed and the generation upon which experiments were performed. For gene-edited lines, describe the editor used, the endogenous sequence targeted for editing, the targeting guide RNA sequence (if applicable) and how the editor was applied.

Authentication

Describe any authentication procedures for each seed stock used or novel genotype generated. Describe any experiments used to assess the effect of a mutation and, where applicable, how potential secondary effects (e.g. second site T-DNA insertions, mosiacism, off-target gene editing) were examined.
