# Supplementary material for: High-depth whole genome sequencing of premalignant breast lesions reveals rearrangement hotspots and personalized management opportunities
Source: Nat Commun. 2026 May 19;17:6931. doi: 10.1038/s41467-026-72952-1 (PMC13389004; doi:10.1038/s41467-026-72952-1)
Supplement: Supplementary file 12 — Transparent Peer Review file [file 41467_2026_72952_MOESM12_ESM.pdf]

nature portfolio

Peer Review File

High-depth whole genome sequencing of premalignant breast lesions reveals rearrangement hotspots and personalized management opportunities

Corresponding Author: Professor Serena Nik-Zainal

This file contains all reviewer reports in order by version, followed by all author rebuttals in order by version.

Version 0:

Reviewer comments:

Reviewer #1

(Remarks to the Author)
Chmelova et al. report a large-cohort, high-depth WGS dataset including 113 paired DCIS and matched-normal samples. The analyses show that although the mutational and copy-number landscapes of DCIS closely resemble those of invasive breast cancer (IBC), signatures of clustered structural variants are intriguingly enriched in DCIS. They further identify SV hotspots (defined as SHOREs) as fragile genomic loci that appear to drive ongoing genome instability in DCIS. Overall, this is an interesting and valuable study. However, I have several comments that I believe would strengthen the manuscript.
1. The enrichment of clustered SV signatures in DCIS relative to IBC/MBC is indeed striking. However, could this be largely due to the much higher sequencing depth in the current DCIS cohort, which would increase sensitivity for detecting clustered SVs? The authors note that high-depth WGS substantially improved detection of ecDNA-derived amplicons compared to previous shallow WGS datasets. Therefore, sequencing coverage needs to be carefully accounted. In addition, computational pipelines may also contribute to the discrepancy. Were the same SV detection and signature-analysis tools used as in the IBC/MBC studies?
2. It would be informative to analyze the clonality of clustered SV events, amplicons or SHOREs. Are many of these events subclonal, arising independently across distinct sublineages within the same lesion? If so, this could provide important insights into the extent of genomic instability and evolution in DCIS. More broadly, is intra-lesion heterogeneity in DCIS higher than in IBC/MBC?
3. While the focus on SVs is a major strength in this paper, the analysis of the natural history and co-evolution of somatic driver mutations and SVs is relatively underdeveloped. Given the high sequencing depth and the scale of this cohort in a premalignant context, elucidating the earliest somatic events and the temporal dynamics of tumor initiation would be particularly impactful.
4. A more open question arises regarding how “precancer” should be defined genomically. DCIS lesions already harbor extensive genomic alterations as this study demonstrated, raising the question of what additional forces drive malignant transformation from DCIS to invasive disease. Addressing this point, even if speculative, would add valuable context.
5. This study generates an extremely important dataset for the community. Unfortunately, the raw sequencing data cannot be made publicly available due to patient-privacy restrictions. If possible, the authors might consider providing controlled-access options, or additional processed data (VCFs for somatic mutations/indels, purity estimates, SVs) to maximize the utility of this resource.

Reviewer #2

(Remarks to the Author)
Review Nature Communications manuscript NCOMMS-25-82013-T

The authors present a high-depth WGS analysis of primarily higher-grade DCIS with the goal of better defining genomic features associated with risk of progression to invasive disease. Most notably, they define specific genomic locations in DCIS that are susceptible to producing initiating lesions for structural variants (SVs) prone to subsequent evolution, or SHOREs. The other sections of the manuscript, however, are largely confirmations of the established literature.
The authors compare WGS differences between DCIS and IBC. But the cohorts are likely skewed with a high proportion of

high grade DCIS (and, not discussed, the sampling bias due to many low grade DCIS being much smaller and therefore harder to profile). The authors need to find some normalization feature that allows for these types of comparisons, otherwise they are not very meaningful.
The authors detail genomic changes in DCIS, but many ideas in the following sections have been previously shown.
1. DCIS has similar drivers, CNAs, and mutations as IBC.
2. Focal amplifications and ecDNA formation are common early events in DCIS and are tightly linked to clustered SVs.
3. Focal amplicons are evolutionary accelerants and dependent on TP53 disruption.
In sections 4 and 5, the authors report that RS1 hotspots can be sites of initiating lesions or breaks for SVs. In addition, they argue that these SVs hotspots can continue to evolve (renamed SHOREs) and form complex focal amplicons at increasing rates from DCIS to MBC. While the mapping of SHOREs in DCIS is novel, these types of hotspots have been reported in the literature.
Finally, in sections 6 and 7, the authors present mechanisms that lead to breaks and rearrangements in SHOREs. They present evidence for both high transcriptional activity and enriched replication origins in SHOREs, which can lead to TRCs and thus increased risk for breaks. The evidence is clear, but these mechanisms are also not novel.
In the closing section 8 and Discussion, the authors point out that 46% of lesions in DCIS had targetable abnormalities and/or carried negative prognostication indices in IBC. These include ERBB2 amplified via ecDNA, HRDetect-high lesions, APOBEC hypermutation, PIK3CA/AKT/PTEN alterations, and NTHL1-driven base excision repair deficiency.
While this clearly highlights the general utility of using WGS in the clinic for DCIS, this section could be more impactful if the authors can provide any context for ongoing or future work on SHOREs and how they may be relevant or contribute to designing personalized treatments in DCIS.

Version 1:

Reviewer comments:

Reviewer #1

(Remarks to the Author)
The authors have done an amazing job and addressed my previous concerns well. It's a solid study that provides important insights into the biology of precancer. I don't have further comments.

Reviewer #2

(Remarks to the Author)
My concerns have been addressed.

Open Access This Peer Review File is licensed under a Creative Commons Attribution 4.0 International License, which permits use, sharing, adaptation, distribution and reproduction in any medium or format, as long as you give appropriate credit to the original author(s) and the source, provide a link to the Creative Commons license, and indicate if changes were made.

In cases where reviewers are anonymous, credit should be given to 'Anonymous Referee' and the source.
The images or other third party material in this Peer Review File are included in the article’s Creative Commons license, unless indicated otherwise in a credit line to the material. If material is not included in the article’s Creative Commons license and your intended use is not permitted by statutory regulation or exceeds the permitted use, you will need to obtain permission directly from the copyright holder.
To view a copy of this license, visit https://creativecommons.org/licenses/by/4.0/

High-depth whole genome sequencing of premalignant breast lesions: Biological insights into rearrangement hotspots and opportunities for personalized management (NCOMMS-25-82013-T)

We would like to thank the reviewers for taking the time to review our manuscript and for their constructive comments, which we believe have helped to improve the manuscript.

Please find below, in blue, our detailed point-by-point response to the reviewers' comments. At the end of the document, we have also listed minor edits to the text and figures of clarifications/corrections that we identified during the revision process.

Reviewer 1:

1. **The enrichment of clustered SV signatures in DCIS relative to IBC/MBC is indeed striking. However, could this be largely due to the much higher sequencing depth in the current DCIS cohort, which would increase sensitivity for detecting clustered SVs? The authors note that high-depth WGS substantially improved detection of ecDNA-derived amplicons compared to previous shallow WGS datasets. Therefore, sequencing coverage needs to be carefully accounted. In addition, computational pipelines may also contribute to the discrepancy. Were the same SV detection and signature-analysis tools used as in the IBC/MBC studies?**

   Thank you for the request on clarification of sequencing depth.

   First, to provide reassurance, the sequencing depths of the DCIS samples are comparable to most of the IBCs and all the MBC samples in the study. Only a subset of IBCs from the ICGC cohort had a lower mean tumour sequencing coverage. We apologise if there was confusion. Perhaps the confusion arises out of the discussion regarding previous DCIS samples. The current DCIS samples are deeper in depth compared to the Houlahan *et al.* study (which was shallow sequencing), but they are perfectly comparable to the IBCs and MBCs in our study.

   For clarity, Table 1 below summarises the sequence coverage statistics of the cohorts used in this study. We have also added this information to the description of these cohorts in the Methods section (Pages 11&12, lines 606-618).

BC stage	Cohort	Sample count	Tumour coverage	Normal coverage
**DCIS**	DCIS	113	104	39
**IBC**	100kGP	2,445	96	36
**IBC**	ICGC	640	43	30

**MBC**	HMF	661	90-100	30-35

Table 1. Mean sequencing tumour and normal coverage in the individual cohorts.

Second, in case there remain concerns, to address the potential impact of sequencing depths on the prevalence of clustered SVs, we show that the proportion of clustered SVs identified per sample is comparable between high depth 100kGP cohort and lower depth ICGC cohort (**Fig. 1**).

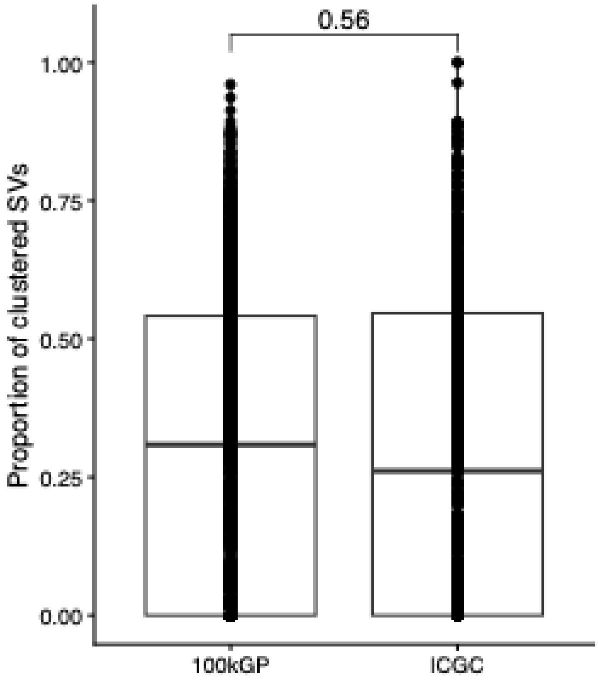

Figure 1. Proportion of clustered SVs out of all SVs identified in high-coverage 100kGP cohort and lower-coverage ICGC cohort.

Third, to assess the impact of sequencing depth on the prevalence of clustered SV signatures, we performed a down-sampling experiment on a subset of DCIS samples (n = 27) to coverage levels of 40× and 30× (**Fig. 2**). We found that the prevalence of clustered SV signatures RS6a and RS6b in DCIS were comparable even at lower sequencing depths. RS4, a translocation-defined signature did show some negative impact at the lowest coverage of 30×. If we consider the IBC samples in the ICGC study that had the lowest mean sequence coverage, this was still at a mean of 42× and well above the lowest depth of the down-sampling exercise.

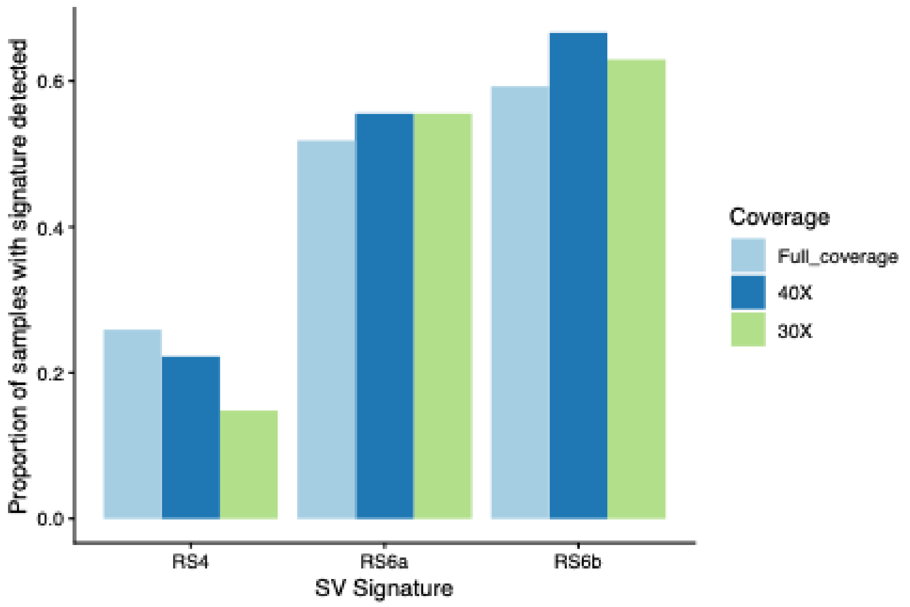

Figure 2. The impact of down-sampling of DCIS samples to 30X and 40X coverage on the SV signature analysis, particularly prevalence of clustered RS4, RS6a and RS6.

In terms of variant callers used for SV detection, we used the Brass variant caller for the DCIS cohort and ICGC cohort. For the 100kGP cohort, we used the variant calls from Black *et al*. (2025) that used Manta (v1.5). For the HMF cohort, SVs were called with Manta (v1.0.3) with default parameters followed by additional filtering using Breakpoint-Inspector v.1.5. Given Manta variant caller was used for the majority of IBC and MBC cohort, we have repeated SV variant calling for the 113 DCIS samples using Manta (v1.6) with default parameters. To take a conservative approach, SVs were filtered for PASS variants with PR >= 8 and removing calls with ‘IMPRECISE’ tag. In fact, the burden of clustered SVs (**Fig. 3**) is higher using Manta compared to the BRASS. This suggests Brass (originally used in DCIS cohort) did not fuel the high SV burden and instead provided a conservative estimate of SV burden.

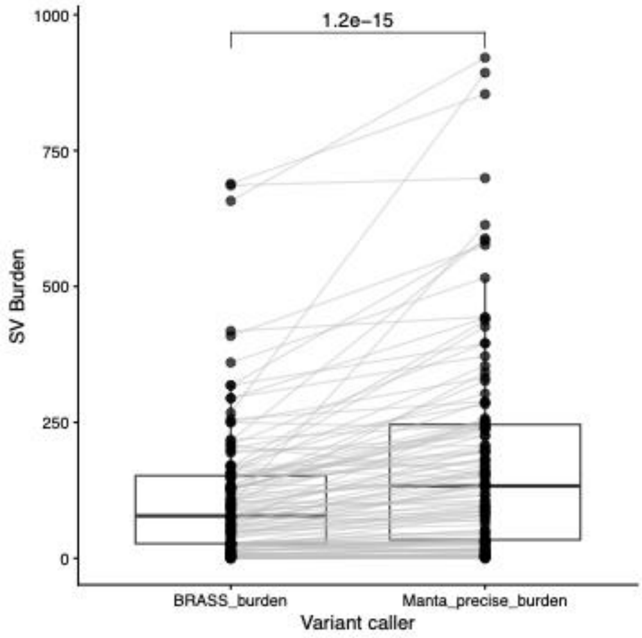

Figure 3. Comparison of the total SV burden in DCIS using BRASS and Manta variant callers.

In terms of fitting mutational signature, analyses for all cohorts were performed using the same algorithms from r-package signature.tools.lib, as outlined in Methods (Page 13, line 663-683).

2. **It would be informative to analyze the clonality of clustered SV events, amplicons or SHOREs. Are many of these events subclonal, arising independently across distinct sublineages within the same lesion? If so, this could provide important insights into the extent of genomic instability and evolution in DCIS. More broadly, is intra-lesion heterogeneity in DCIS higher than in IBC/MBC?**

Thank you for this very interesting comment. To assess clonality states of individual SHOREs, we identified all SHOREs that had a SV in DCIS - 250 such occurrences were identified in 74 samples. The clonality states of the 250 regions were estimated using MutationTimeR algorithm inferred based on variant allele fractions of SNVs within the region. The clonal fraction of SNVs in each of the 250 occurrences are presented in a heatmap below (**Fig. 4**), to understand clonal states for each SHORE across all the DCIS patients.

We found:

- That the majority of these 250 occurrences are clonal: 59.2% (n = 148) (**Fig. 4**).

- 10 SHOREs were always clonal, including chr17_37.7mb (*ERBB2*), suggesting an early evolutionary role.
- No SHORE regions were exclusively subclonal.

Indeed, the most prominent finding offered by the heatmap is that subclonality, if present, was largely determined by individual sample characteristics rather than by the SHORE. While a subset of DCIS cases (n = 27) showed all SHOREs as early clonal origin, eleven samples demonstrated a greater subclonal activity in many SHOREs, with >50% of active shores being predominately sub-clonal. For instance, sample LP2101771 harboured mostly subclonal SNVs (>50%) in 4/6 SHOREs, suggesting that the sample has multiple distinct sub-lineages. This is a feature of the sample however, and not a SHORE-determined factor.

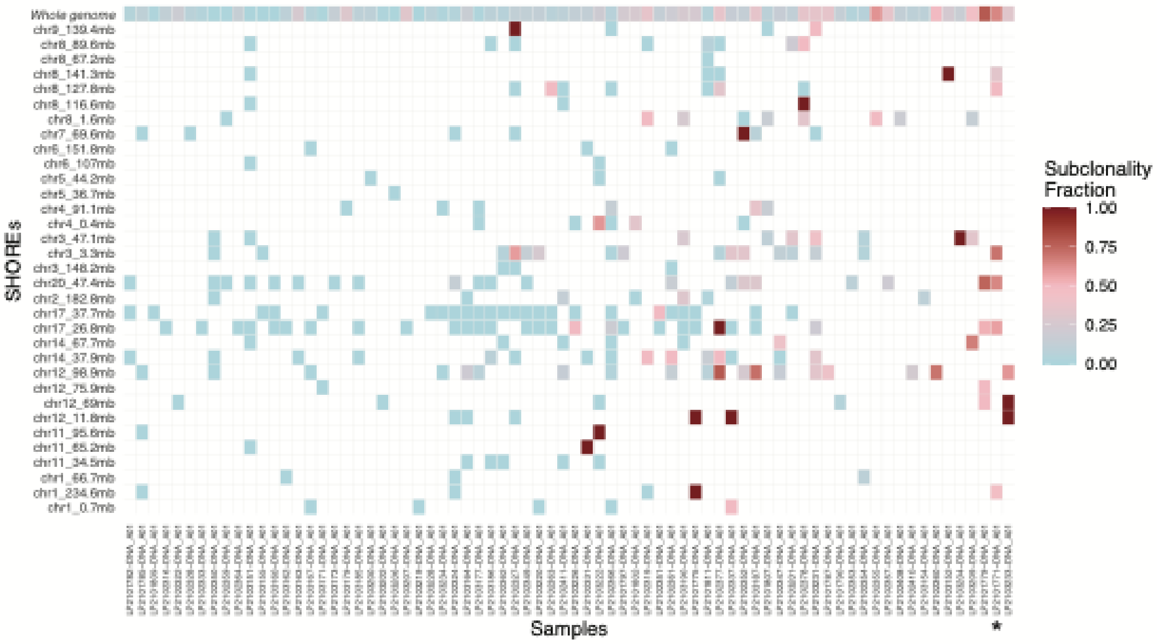

Figure 4. Summary of subclonality within active SHOREs with a SV present (n = 250). The samples were ordered by the number of clonal SHOREs per sample and the number of largely subclonal SHOREs. The SHOREs were ordered by a proportion of clonal SHOREs. Asterisk indicates sample LP2101771.

We thank the reviewer for raising this point and have included the following sentences in the main text (Page 7, line 386-389): ‘Finally, we examined the clonal characteristics of the SVs in SHOREs across the patient cohort to find that ten SHOREs including chr17_37.7mb (ERBB2) harbored only clonal characteristics (Fig. S6A) and no SHOREs were found to be exclusively subclonal. These results reinforce how SVs at SHOREs are early contributors to tumor evolution.’ The figure was added as a supplementary figure 6A. We

have also included a description of the method used in the Methods section (Page 15, Lines 771-776).

3. **While the focus on SVs is a major strength in this paper, the analysis of the natural history and co-evolution of somatic driver mutations and SVs is relatively underdeveloped. Given the high sequencing depth and the scale of this cohort in a premalignant context, elucidating the earliest somatic events and the temporal dynamics of tumor initiation would be particularly impactful.**

   Thank you for the critique. As noted above, most SHOREs are in fact clonal, limiting our ability to perform co-evolutionary analyses and to draw strong conclusions from it. Nevertheless, to evaluate the possibility of performing a co-evolution analysis of somatic driver mutations with SVs, we investigated the clonality states of driver events. We applied the MutationTimeR algorithm on substitution somatic drivers. The analysis showed that 8.9% of DCIS drivers were subclonal, including *TP53* (*n* = 2), *PIK3CA* (*n* = 2), and *FOXA1* (*n* = 2). First, this is a negligible difference compared to the IBC cohort, where 6.9% of drivers were subclonal (Black *et al.*, 2025). Second, at these low numbers (single digits), it would be impossible to draw strong conclusions about co-evolution of somatic drivers and SVs.

   Additionally, the analysis of SNV clonality showed that in 74 samples analysed in Fig.4, less than 5% of samples (n = 3) are predominantly subclonal (>50% of SNVs are subclonal).

4. **A more open question arises regarding how “precancer” should be defined genomically. DCIS lesions already harbor extensive genomic alterations as this study demonstrated, raising the question of what additional forces drive malignant transformation from DCIS to invasive disease. Addressing this point, even if speculative, would add valuable context.**

   Thank you for the suggestion. An extremely interesting point and one we cannot answer with this manuscript. Thus, we have included it in our limitations section.

   We included the following sentence in the manuscript (Page 10, line 550-554): ‘Intriguingly, the extent of genomic instability observed even in pre-malignant lesions does raise a pertinent question – what is the tipping point at which a genomically-abnormal lesion truly becomes malignant? To address this question, we will indeed require a longitudinal view reaching beyond genomics into multimodal integration with (at least) the epigenetic and immunological spheres.’

5. **This study generates an extremely important dataset for the community. Unfortunately, the raw sequencing data cannot be made publicly available due to patient-privacy restrictions. If possible, the authors might consider providing controlled-access options, or additional processed data (VCFs for somatic mutations/indels, purity estimates, SVs) to maximize the utility of this resource.**

   Thank you. We agree that this is potentially a very useful data set for the community. We have therefore, deposited as much of the data as we are able under the patient-privacy restrictions of the specific collection studies. All links and accession numbers have now been added to the Data and materials availability section of the paper (Page 17, lines 872-885). This included the following:

   - Raw WGS data has been deposited in EGA for a total of 77 tumour/normal pairs. 26 of these pairs have restrictions to be used for somatic mutation calling only. Accession numbers: EGAD50000002071 https://ega-archive.org/datasets/EGAD50000002071 EGAD50000002237 https://ega-archive.org/datasets/EGAD50000002237
   - RNA sequencing data for the 77 samples which have not previously been published has now been deposited in EGA. Accession number: EGAD50000002123
   - In addition, the filtered somatic mutation calls for all samples included in this study have been deposited in Mendeley data (https://doi.org/10.17632/h8fv2tc8d4.1)
   - Information on the purity estimates were added to the supplementary table 1.
   - Mutational profiles and signatures of all DCIS samples can be viewed at our website: https://signal.mutationalsignatures.com/explore/main/cancer/signatures?mutationType=1&study=13.

Reviewer 2

1. **The authors present a high-depth WGS analysis of primarily higher-grade DCIS with the goal of better defining genomic features associated with risk of progression to invasive disease. Most notably, they define specific genomic locations in DCIS that are susceptible to producing initiating lesions for structural variants (SVs) prone to subsequent evolution, or SHOREs. The other sections of the manuscript, however, are largely confirmations of the established literature.**

The authors compare WGS differences between DCIS and IBC. But the cohorts are likely skewed with a high proportion of high grade DCIS (and, not discussed, the sampling bias due to many low grade DCIS being much smaller and therefore harder to profile). The authors need to find some normalization feature that allows for these types of comparisons, otherwise they are not very meaningful.

Thank you for comments. While we already address the bias towards high-grade lesions in cohort, we clarified the sentences (Page 4, lines 209-214) in the text to mention the limitations of sufficient DNA extractions from small low-grade lesions. 'Compared to other reported DCIS populations (14–16), this cohort (**Fig. 1**) was skewed towards high-grade lesions (63.7% versus 47.0–51.5%) due to limited available material for DNA extraction from low-grade lesions which tend to be smaller in size. This cohort also contains a higher proportion of younger patients (<56 years, 56.6% versus 31.8%) and HER2-positive lesions (53.1% versus 34.4%) **(Fig. S1)**'.

Additionally, in the discussion (Page 10, lines 541-545) we previously addressed the skewed nature of the cohort as a potential limitation: 'The skew towards more "aggressive" lesions is not representative of the general DCIS landscape. However, these are the lesions that represent the clinical conundrum, where surgical intervention based on size, grade or multifocality occurs, but for which subsequent clinical management remains unclear.'

While we appreciate the suggestion for a normalisation feature, most cancer WGS genomic studies report the skewed cohort as a limitation of the cohort rather than attempting to apply any normalisation features. Additionally, any normalisation for grade would require making assumptions about the genetic landscape of low-grade samples we simply do not have, and thus, would be speculative and potentially misleading.

2. **The authors detail genomic changes in DCIS, but many ideas in the following sections have been previously shown.**
   1. DCIS has similar drivers, CNAs, and mutations as IBC.
   2. Focal amplifications and ecDNA formation are common early events in DCIS and are tightly linked to clustered SVs.
   3. Focal amplicons are evolutionary accelerants and dependent on TP53 disruption.

   Thank you. One of the strengths of our study is the unprecedented size of this high-depth WGS DCIS cohort. Our study has allowed the most comprehensive study of the genomic landscape of DCIS to date. While describing this genomic landscape (and we would be expected to do so), it is important for us to

reinforce past findings. That lends credence to past work and provides reassurance about our work too. We do not overclaim – indeed, where relevant, we simply state overtly where we are reinforcing past findings (for example, page 5, lines 255-257 comparing DCIS and IBC drivers).

Advances over past work are stated where appropriate. For example, while it is true that ecDNA formation has been previously identified as an early event in DCIS, the previous literature reported only a small subset (~8%) of DCIS samples harboured ecDNA (Houlahan *et al*., 2025). The strength of this study using high depth WGS, is that it identifies ecDNA at greater scale, particularly in HER2-positive samples where ecDNAs were observed in 48% of cases.

The high prevalence of clustered SVs in DCIS in itself is a new observation and while the link to the ecDNA may be obvious, it serves as additional validation for ecDNA. It also underscores how amplicon-formation are not driven solely by ecDNA.

Additionally, we put our observations on TP53 into perspective with the other literature (in the amended sentence on page 6 lines 310 – 313): '*TP53* pathway disruption, including by TP53-repressors *PPM1D*, *MDM2*, and *MDM4*, was associated with amplicon presence (P<0.0001, Fisher's exact test), broadly in agreement with previous observations (Luebeck *et al*., 2023)'.

3. **In sections 4 and 5, the authors report that RS1 hotspots can be sites of initiating lesions or breaks for SVs. In addition, they argue that these SVs hotspots can continue to evolve (renamed SHOREs) and form complex focal amplicons at increasing rates from DCIS to MBC. While the mapping of SHOREs in DCIS is novel, these types of hotspots have been reported in the literature.**
Finally, in sections 6 and 7, the authors present mechanisms that lead to breaks and rearrangements in SHOREs. They present evidence for both high transcriptional activity and enriched replication origins in SHOREs, which can lead to TRCs and thus increased risk for breaks. The evidence is clear, but these mechanisms are also not novel.

Thank you for this comment. We agree that transcription–replication conflicts and replication-associated breakage are well-established mechanisms of genome instability and are not presented here as novel per se.

The novelty of our analysis lies in the investigation of these phenomena in high-coverage WGS DCIS, which enables us to identify SHOREs at an early, pre-invasive stage and to characterise their biological properties prior to extensive clonal evolution. Importantly, SHOREs were defined solely based on SV breakpoint patterns, independent of transcriptional data.

The observed enrichment for high transcriptional activity therefore serves as independent functional validation that SHOREs correspond to biologically active genomic regions already under stress in DCIS. This demonstrates that these loci are not merely computational SV hotspots or generic fragile sites, but regions that are transcriptionally engaged early and thus susceptible to recurrent breakage.

Furthermore, the presence of elevated transcription in DCIS SHOREs supports a model in which early transcription-associated stress contributes to initiating lesions that subsequently undergo continued rearrangement and amplification during progression to invasive and metastatic disease.

4. **In the closing section 8 and Discussion, the authors point out that 46% of lesions in DCIS had targetable abnormalities and/ or carried negative prognostication indices in IBC. These include ERBB2 amplified via ecDNA, HRDetect-high lesions, APOBEC hypermutation, PIK3CA/AKT/PTEN alterations, and NTHL1-driven base excision repair deficiency.**
   While this clearly highlights the general utility of using WGS in the clinic for DCIS, this section could be more impactful if the authors can provide any context for ongoing or future work on SHOREs and how they may be relevant or contribute to designing personalized treatments in DCIS.

   We appreciate the suggestions and have incorporated the following statement into the discussion (Page 10, lines 527-529): ….'However, longitudinal studies are required to understand whether structural variants in SHORES could serve as a biomarker and harbinger of recurrence as IBC.'

Additionally, we have made following changes:

- In Supplementary figures S3A,B (right), InD22 was renamed to InD17 and pgel_Breast k=11, Cluster 11, U1 was renamed to InD30, in agreement with the most recent published nomenclature (Black *et al*., 2025). InD22 was also renamed as InD17 in Supplementary table 3.
- In Figure 3, Fig. 3E the circus plot for MBC was updated to show all chromosomes that harbour SHORE regions to provide a fair comparison with the circus plots for DCIS and IBC cohorts.
- The numbering of supplementary figures changes, as a new supplementary figure was created that show the clonality in SHOREs.
  - Briefly:
    - S6A – a new figure
    - S5E,F → S6B,C
    - S6 → S7

- In Methods (Page 14, line 708), we removed repeated explanation of the abbreviations HER2 and ER.
- Page 5 Line 282 - HER2 corrected to ERBB2
